# Modeling study of the effect of placebo and medical therapy on storage and voiding symptoms, nocturia, and quality of life in men with prostate enlargement at risk for progression

**DOI:** 10.1038/s41391-023-00731-w

**Published:** 2023-10-04

**Authors:** Stavros Gravas, Juan Manuel-Palacios, Chandrashekhar Chavan, Claus G. Roehrborn, Matthias Oelke, Marcio Augusto Averbeck, Arunangshu Biswas, Llenalia María García, Khadeeja Mohamed, Vanessa Cortes

**Affiliations:** 1 Department of Urology, Medical School, University of Cyprus, Nicosia, Cyprus; 2 Global Medical Urology, GSK, Madrid, Spain; 3 Global Medical Urology, GSK, Mumbai, India; 4 Department of Urology, University of Texas Southwestern Medical Center, Dallas, TX USA; 5 Department of Urology, St. Antonius-Hospital, Gronau, Germany; 6 Department of Urology, Moinhos de Vento Hospital, Porto Alegre, Brazil; 7 Development Biostatistics, GSK, Bengaluru, India; 8 Advanced Analytics, Access Consulting Parexel, Malaga, Spain; 9 Development Biostatistics, GSK, Brentford, UK; 10 Global Medical Urology, GSK, Bogota, Colombia

**Keywords:** Prostatic diseases, Medical research

## Abstract

**Background:**

Modeling studies using large datasets from men with lower urinary tract symptoms/benign prostate enlargement (LUTS/BPE) can predict changes in International Prostate Symptom Score (IPSS) and risk of acute urinary retention/surgery under different treatment regimens and according to predictors (baseline characteristics) that commonly define risk of progression. We assessed the impact of treatments on different symptom types (storage, voiding, and nocturia), quality of life (QoL; IPSS Q8), and BPH Impact Index [BII]).

**Methods:**

Generalized least squares models were used to predict each outcome. Data from the CombAT study were used to predict outcomes for active treatments (dutasteride, tamsulosin, combination therapy). Predictors included: age; IPSS total, storage, voiding, nocturia and QoL (IPSS Q8) scores; BII; prostate volume; maximum urine flow rate (Qmax), prostate-specific antigen, postvoid residual urine (PVR); alpha-blocker usage within 12 months. Data from phase III dutasteride monotherapy studies were used to predict placebo outcomes. Results were visualized using an interactive web-based tool (www.bphtool.com).

**Results:**

Combination therapy provided greater predicted benefit than either monotherapy for all five outcomes for most patient profiles within the CombAT inclusion criteria. PVR and corresponding subscores were significant predictors of change in both storage and voiding subscores. Alpha-blocker use within 12 months, age (storage subscore), and Qmax (voiding subscore) were also significant predictors. PVR, age, Qmax, and nocturia score were significant predictors of change in nocturia. PVR, Qmax, previous alpha-blocker use, total IPSS, and QoL (IPSS Q8) score were significant predictors of change in QoL (IPSS Q8) score. For BII, significant predictors were PVR, age, total IPSS, and BII score. The multivariable effect of covariates and treatments is best visualized through the interactive web-based tool.

**Conclusions:**

This predictive modeling study informs our understanding of how risk factors for disease progression interact and affect treatment impact on different symptom types and QoL scores.

## Introduction

Clinical guidelines provide clear recommendations for assessing men with lower urinary tract symptoms (LUTS) as a result of benign prostatic enlargement (BPE), and have two main objectives: to establish a differential diagnosis (since the origin of LUTS in men is multifactorial) and to define the clinical profile (including risk of disease progression, e.g., higher prostate volume [PV], higher serum prostate-specific antigen [PSA] concentration, advanced age, higher post-void residual volume [PVR], and lower urinary flow) of men with LUTS in order to provide personalized and appropriate care [1, 2].

Men with LUTS/BPE can be affected by voiding, storage, and post-micturition symptoms [3]. Voiding symptoms are thought to arise from urethral obstruction, often as a result of BPE [4, 5]. Storage symptoms (including nocturia) are often seen in men with overactive bladder (OAB) and BPE, and result from altered smooth muscle structure and function in the bladder and prostate [5, 6]. Voiding and storage symptoms cause bother and impair quality of life (QoL) [7–9]. Nocturia is among the most frequently reported and bothersome symptom [10, 11] and the major cause of physician consultations in men with LUTS/BPE [12]. LUTS/BPE can progress to worsening of symptoms and complications, such as acute urinary retention (AUR) and BPE-related surgery. Therefore, a treatment approach that targets the underlying disease mechanism and improves different symptom types, as well as reducing the risk of AUR/surgery, would be advantageous.

The wealth and complexity of clinical trial data make it difficult to understand how evidence can optimally inform personalized treatment decisions, since results are typically presented as mean values for a defined population. Insights from the application of predictive analytics to large clinical cohorts can support physicians in understanding the key characteristics and relationships behind responses to individual treatments. This knowledge can help to tailor the treatment approach in patients with LUTS/BPE at different levels of progression risk. Visualizing individual risks, and how they may change in the future depending on treatment choice (active or placebo), may support healthcare professionals and patients in making more personalized, data-driven decisions based on easily accessible baseline parameters and risk factors.

A predictive analytics solution, using large datasets of patients receiving placebo, dutasteride, tamsulosin, or dutasteride/tamsulosin combination therapy (CT), was developed to project the change from baseline in total IPSS and risk of AUR or surgery under these different treatment regimens, according to baseline characteristics that commonly define patients at risk of disease progression, such as age ≥50 years, moderate-to-severe LUTS, PV ≥ 30 mL, and PSA ≥ 1.5 ng/mL [13]. The study showed that the vast majority of patients benefit more from dutasteride or dutasteride/tamsulosin CT compared with tamsulosin alone, highlighting the prognostic importance of baseline covariates to predict treatment response in individual profiles [13]. An educational, interactive web-based tool was developed to facilitate visualization and understanding of predicted outcomes for any individual profile meeting the CombAT study entry criteria, integrating any possible combination of variables (predictors). The tool is available at www.bphtool.com.

The current study aimed to expand on the predictive analytics solution described above to better understand how placebo, dutasteride, tamsulosin, or CT impact different symptom types (IPSS subscores for storage, voiding, and nocturia [IPSS Q7]) over time, as well as QoL (IPSS Q8) and BPH Impact Index (BII), in different profiles of men with BPE at risk of progression as defined by their baseline characteristics. The web-based tool was updated to facilitate visualization and understanding of study results.

## Methods

### Data sources, endpoints, and variables

Datasets are the same as those used in the initial predictive analytics solution [13]; three placebo-controlled dutasteride trials (ARIA3001, ARIA3002, and ARIB3003) and one trial comparing the active therapies: dutasteride, tamsulosin, and combination (ARI40005; CombAT) [14, 15]. Reasons for not including other studies of dutasteride and finasteride have been previously described [13].

Using clinically relevant baseline characteristics that commonly define patients with LUTS/BPE at risk of disease progression, we aimed to separately predict longitudinal change from baseline in (i) IPSS voiding subscore, (ii) IPSS storage subscore, (iii) IPSS nocturia subscore (Q7), (iv) QoL (IPSS Q8), and (v) BII score. Definitions of the disease outcomes explored, and duration of follow-up, are detailed in the Supplementary information (Table S1). The covariates used to predict treatment effect on LUTS/BPE outcomes in these models are shown in Table 1.

**Table 1:** Covariate use in models to predict treatment effect on LUTS/BPE outcomes.

Covariate	Range for covariate	LUTS/BPE outcome and predictors used						
		This study					Gravas et al. [13]	
		IPSS-S	IPSS-V	IPSS-N	IPSS-Q	BII	Total IPSS	AUR/S
Age (years)	50–100	YES	YES	YES	YES	YES	YES	YES
Total IPSS	12–35	NO	NO	NO	YES	YES	YES	YES
IPSS-S	0–15	YES	NO	NO	NO	NO	NO	NO
IPSS-V	0–20	NO	YES	NO	NO	NO	NO	NO
IPSS-N	0–5	NO	NO	YES	NO	NO	NO	NO
IPSS Q	0–6	NO	NO	NO	YES	NO	NO	NO
BII	0–13	NO	NO	NO	NO	YES	NO	NO
PSA (ng/mL)	1.5–10	YES	YES	YES	YES	YES	YES	YES
PV (mL)	30–150	YES	YES	YES	YES	YES	YES	YES
Qmax^a^ (mL/s)	6–15	YES	YES	YES	YES	YES	YES	YES
PVR (mL)	0–250	YES	YES	YES	YES	YES	YES	YES
AB use^b^	Yes/no	YES	YES	YES	YES	YES	YES	YES
Randomized treatment^c^	Yes/no	YES	YES	YES	YES	YES	YES	YES

Age is recorded at the start of treatment. All other covariates are recorded at baseline.

The baseline total IPSS is not used as a covariate in the prediction of IPSS storage and voiding sub scores as well as IPSS Q7 nocturia, since the total IPSS scores subsume the above subscore showing a high correlation.

*AB* alpha-blocker, *AUR* acute urinary retention, *BII* BPH impact index overall score, *BPE* benign prostate enlargement, *BPH* benign prostatic hyperplasia, *IPSS* international prostate symptom score, *IPSS-N* IPSS Q7 nocturia subscore, *IPSS-Q* IPSS Q8 quality of life score, *IPSS-S* IPSS storage subscore, *IPSS-V* IPSS voiding subscore, *LUTS* lower urinary tract symptoms, *PSA* prostate-specific antigen, *PV* prostate volume, *PVR* post-void residual volume, *Qmax* maximum urinary flow rate, *S* surgery.

^a^With minimum voided volume >125 mL at baseline.

^b^Within the last 12 months.

^c^Placebo, dutasteride, tamsulosin, or dutasteride/tamsulosin combination therapy.

### Predictive modeling strategy

Patient-level data from the CombAT study [15] were used in a model to predict outcomes with active treatments (dutasteride, tamsulosin, and CT). Patient-level data from three phase III dutasteride monotherapy studies [14] were used in a model to predict outcomes with placebo.

A generalized least squares model was used to predict each of the five disease outcomes, assuming all outcomes to be continuous. Models were validated using a 10-fold cross-validation method, and performed equally well in the training and test sets. Model evaluation was compared before and after excluding non-relevant predictors and nugatory predictors, based on estimated parameters with associated *p* values > 0.05 and insignificant improvement of model when its presence was excluded. After exclusion, the change in parameter estimates for the remaining predictors was evaluated; those with a relative change in estimate >30% were considered possible confounders and adjusted for in the final model. The analyses adhere to the Transparent Reporting of a multivariable prediction model for Individual Prognosis Or Diagnosis statement [16].

Table 2 shows summary statistics of baseline characteristics from the CombAT and phase III dutasteride studies that were considered for model development and validation. Additional information on datasets (Table S2), endpoints, variable selection, handling of missing data, modeling strategy, and model performance is provided in the Supplementary material.

**Table 2:** Summary statistics for the baseline characteristics in the BPH studies considered for model development and validation.

Variable	Model	Treatment	Median (IQR)	Mean	SD
Age at treatment start (years)	CombAT	Combination	66.0 (61–71)	66.0	7.0
		Dutasteride	66.0 (61–71)	66.0	7.9
		Tamsulosin	66.0 (61–71)	66.2	7.0
	Placebo	Dutasteride	67 (61–72)	66.5	7.6
		Placebo	66 (61–71)	66.1	7.4
Baseline prostate volume (mL)	CombAT	Combination	48.9 (39.2–63.2)	54.7	23.5
		Dutasteride	48.4 (38.5–63.2)	54.6	23
		Tamsulosin	49.6 (38.7–65)	55.8	24.2
	Placebo	Dutasteride	48.7 (38.5–63.1)	54.9	23.9
		Placebo	48.3 (39–62.3)	54.0	21.9
Baseline post-void residual volume (mL)	CombAT	Combination	50 (20–96)	68.2	66.1
		Dutasteride	50 (20–100)	67.4	63.5
		Tamsulosin	50 (20–100)	67.7	65.2
	Placebo	Dutasteride	56.0 (20–112)	76.6	75.0
		Placebo	59.0 (20–109)	74.9	69.6
Baseline Qmax (mL/s) with minimum voiding volume >125 mL	CombAT	Combination	10.6 (8.4–12.8)	10.9	3.6
		Dutasteride	10.3 (8.0–12.7)	10.6	3.6
		Tamsulosin	10.3 (8.0–12.6)	10.6	3.6
	Placebo	Dutasteride	9.9 (7.6–12.2)	10.1	3.5
		Placebo	10.2 (7.9–12.5)	10.3	3.6
Baseline PSA (ng/mL)	CombAT	Combination	3.4 (2.4–5.1)	4.0	2.1
		Dutasteride	3.4 (2.3–5.1)	3.92	2.1
		Tamsulosin	3.5 (2.4–5.2)	4.0	2.1
	Placebo	Dutasteride	3.4 (2.3–5.3)	4.0	2.1
		Placebo	3.5 (2.3–5.2)	4.0	2.1
Baseline total IPSS	CombAT	Combination	16 (12–21)	16.6	6.3
		Dutasteride	16 (12–20)	16.4	6.0
		Tamsulosin	16 (12–20)	16.4	6.1
	Placebo	Dutasteride	17 (13–21)	17.1	6.0
		Placebo	17 (13–21)	17.2	6.1
Baseline IPSS storage subscore	CombAT	Combination	7.0 (5.0–9.0)	7.3	3.0
		Dutasteride	7.0 (5.0–9.0)	7.2	2.9
		Tamsulosin	7.0 (5.0–9.0)	7.2	2.9
	Placebo	Dutasteride	8.0 (5.0–10.0)	7.7	3.0
		Placebo	8.0 (6.0–10.0)	7.8	3.0
Baseline IPSS voiding subscore	CombAT	Combination	9.0 (6.0–12.0)	9.3	4.3
		Dutasteride	9.0 (6.0–12.0)	9.2	4.3
		Tamsulosin	9.0 (6.0–12.0)	9.2	4.2
	Placebo	Dutasteride	9.0 (7.0–12.0)	9.4	4.1
		Placebo	9.0 (6.0–12.0)	9.4	4.3
Baseline IPSS Q7 nocturia	CombAT	Combination	2 (2–3)	2.4	1.2
		Dutasteride	2 (2–3)	2.4	1.2
		Tamsulosin	2 (2–3)	2.4	1.2
	Placebo	Dutasteride	2 (2–3)	2.4	1.2
		Placebo	2 (2–3)	2.4	1.2
Baseline BPH Impact Index Score	CombAT	Combination	5.0 (3.0–8.0)	5.3	3.1
		Dutasteride	5.0 (3.0–8.0)	5.3	3.0
		Tamsulosin	5.0 (3.0–7.0)	5.3	3.1
	Placebo	Dutasteride	4.0 (2.0–6.0)	4.1	2.7
		Placebo	4.0 (2.0–6.0)	4.0	2.8
Baseline IPSS Q8 QoL	CombAT	Combination	4 (3–5)	3.3	0.9
		Dutasteride	4 (3–5)	3.3	0.9
		Tamsulosin	4 (3–5)	3.3	0.9
	Placebo	Dutasteride	-	-	-
		Placebo	-	-	-

The total N for CombAT study (ARI40005) models is 4841: combination (*n* = 1609), dutasteride (*n* = 1623), tamsulosin (*n* = 1609).

The total N for placebo study (ARIA3001, ARIA3002, ARIB3003) models is 4325: dutasteride (*n* = 2167), placebo (*n* = 2158).

Sample size (N) may be different across the different statistics due to missing values.

Baseline IPSS Q8 QoL data were not included in placebo studies.

*BPH* benign prostatic hyperplasia, *IPSS* international prostate symptom score, *IQR* interquartile range, *Qmax* maximum urinary flow rate, *QoL* quality of life, *SD* standard deviation.

## Results

### Descriptive analysis of baseline variables

A total of 9166 subjects with LUTS/BPE at risk of progression who were included in the CombAT study and placebo-controlled dutasteride monotherapy studies were considered. There were no significant differences in baseline covariates between groups in CombAT or the placebo-controlled dutasteride studies (Tables S3 and  S4). Table 3 presents the relevant covariates with significant estimates (*p* < 0.05) for each of the outcomes.

**Table 3:** Summary of baseline covariates with significant estimates that predict changes in the outcomes analyzed per model.

	CombAT model					Placebo model			
Covariates	Storage	Voiding	Nocturia	Q8 QoL	BII	Storage	Voiding	Nocturia	BII
Main effect of baseline covariates and treatments [(DUT, TAM vs CT), (PBO vs DUT)]									
Age (years)	0.019***	NO	0.018***	NO	−0.009*	0.033**	NO	0.005**	NO
IPSS total score	-	-	-	0.021***	0.017**	-	-	-	0.047***
IPSS storage subscore	−0.603***	-	-	-	-	NO	-	-	-
IPSS voiding subscore	-	−0.652***	-	-	-	-	NO	-	-
IPSS Q7 nocturia subscore	-	-	−0.595***	-	-	-	-	−0.539***	-
IPSS Q8 QoL score	-	-	-	−0.800***	-	-	-	-	-
BII overall score	-	-	-	-	−0.575***	-	-	-	NO
PSA (ng/mL)	NO	0.075*	NO	NO	NO	−0.052**	−0.051*	−0.018**	−0.032*
Qmax (mL/s)	NO	−0.042**	−0.008**	−0.022*	NO	NO	NO	NO	NO
PVR (mL)	0.001**	0.003***	0.001**	0.001**	0.001*	NO	NO	NO	NO
AB = yes	0.259**	NO	NO	0.076*	NO	0.489***	0.553***	NO	0.208**
Treatment (DUT)	0.508*	0.942*	0.660**	0.690*	0.694***	-	-	-	-
Treatment (TAM)	NO	−1.044**	NO	NO	NO	-	-	-	-
Treatment (PBO)	-	-	-	-	-	NO	−1.000***	−0.151*	−0.613***
Interaction effect of baseline covariates with treatment (TAM or DUT) or PBO, and baseline response variables with covariates and treatment or PBO									
PV with TAM	NO	0.017***	NO	0.004*	NO	-	-	-	-
PSA with DUT	NO	−0.121*	NO	NO	NO	-	-	-	-
PSA with TAM	NO	−0.109*	NO	NO	NO	-	-	-	-
PV with PBO	-	-	-	-	-	0.010***	0.016***	0.003**	0.009***
Age with DUT	NO	NO	−0.009**	NO	NO	-	-	-	-
Age with TAM	NO	NO	NO	−0.010*	NO	-	-	-	-
AB = Y with TAM	NO	NO	0.172**	NO	0.462**	-	-	-	-
IPSS storage subscore with DUT	0.053*	-	-	-	-	NO	-	-	-
IPSS storage subscore with age	NO	-	-	-	-	−0.004*	-	-	-
IPSS storage subscore with PVR	NO	-	-	-	-	<0.001*	-	-	-
IPSS voiding subscore with AB = Y	-	0.056*	-	-	-	-	NO	-	-
IPSS voiding subscore with DUT	-	0.085**	-	-	-	-	NO	-	-
IPSS voiding subscore with TAM	-	0.062*	-	-	-	-	NO	-	-
IPSS voiding subscore with PVR	-	NO	-	-	-	-	<0.001*	-	-
IPSS voiding subscore and age	-	NO	-	-	-	-	−0.004**	-	-
IPSS Q7 nocturia subscore with PVR	-	-	−0.001***	-	-	-	-	NO	-
IPSS Q7 nocturia subscore with AB = yes	-	-	NO	-	-	-	-	0.079**	-
IPSS Q8 QoL score with Qmax	-	-	-	0.006*	-	-	-	-	-
IPSS Q8 QoL score with DUT	-	-	-	0.077**	-	-	-	-	-
BII overall score with age	-	-	-	-	NO	-	-	-	−0.005***
BII overall score with PVR	-	-	-	-	NO	-	-	-	<0.001**

“-” indicates that the covariate was not used in the prediction model for that outcome.

“NO” indicates *p* > 0.05; therefore, estimate value not included.

The reference treatment is CT (CombAT models) for DUT and TAM arm comparisons and DUT (placebo models) for PBO arm comparison.

Covariates that showed no significant estimates for any outcome explored are not included in this summary table. Complete details for all covariates and estimates (significant or not) for all outcomes and models can be found in the Supplementary material.

*p = 0.05–0.01; **p = 0.01–0.001; ***p < 0.001 (*p*-values have not been adjusted for multiplicity).

*AB* alpha-blocker, *BII* BPH impact index overall score, *BPH* benign prostatic hyperplasia, *CT* combination treatment, *DUT* dutasteride, *IPSS* international prostate symptom score, *PBO* placebo, *PSA* prostate-specific antigen, *PVR* post-void residual volume, *Qmax* maximum urinary flow rate, *QoL* quality of life, *TAM* tamsulosin.

Examples for interpretation: (1) Higher baseline IPSS storage subscore is a significant covariate to predict storage symptom improvement in all treatments (coefficient of –0.603; *p* < 0.001). However, higher IPSS storage subscore is a significant covariate to predict superior improvement in storage symptoms with CT vs DUT (interaction effect 0.053; *p* = 0.04). (2) DUT provides a change from baseline in IPSS storage subscore that is 0.508 times higher than CT (less improvement). (3) Significant estimates from covariates with non-significant interaction effect are interpreted directly, e.g., storage CombAT model, age (coefficient 0.019; *p* < 0.001) has no significant interaction term; therefore, it is subject to direct interpretation: a 1-year increase causes an increase (worsening) in the change from baseline of the IPSS storage subscore of 0.019 units.

### IPSS storage subscore

**Fig. 1:**
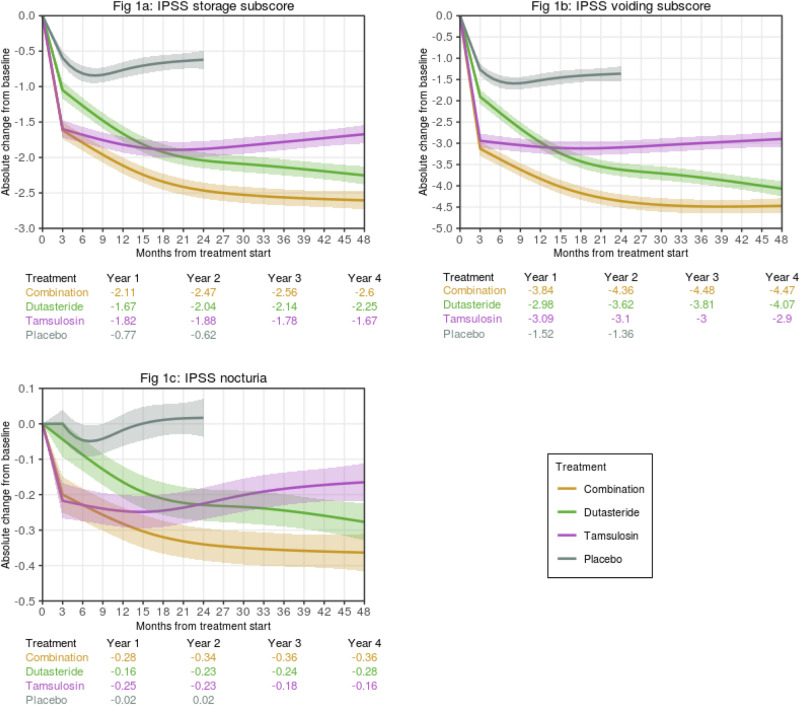
Examples of estimated trajectories over time of absolute change from baseline in IPSS subscores with 95% confidence intervals, by treatment group. **a** storage subscore, **b** voiding subscore, **c** nocturia subscore. **Obtained from** www.bphtool.com. Estimates for placebo are made using a model trained on monotherapies data only (2-year study duration). These examples consider mean values of covariates considered to estimate the trajectories for both treatments and placebo for the intended outcomes: (1) Common predictors for all outcomes: age, 66 years; PV, 55 mL; PVR, 68 mL; Qmax, 11 mL/s; PSA, 4 ng/mL; alpha-blocker use, no. (2) Additional outcome-specific predictors: IPSS storage subscore, 7; IPSS voiding subscore, 9; IPSS nocturia (Q7) score, 2.

The benefit of treatment (reduction in storage subscore) is predicted to be greater with dutasteride plus tamsulosin than with either monotherapy for most patient profiles within the CombAT study inclusion criteria. Figure 1a shows the change from baseline in IPSS storage subscore for an example patient profile, based on mean values of the relevant covariates considered in this model, as visualized on the updated interactive tool (www.bphtool.com).

The baseline covariates of PVR, previous alpha-blocker use, age, and baseline storage subscore were significant predictors of the change in storage subscore in the CombAT model (Table 3, Table S6). The positive sign of the coefficient indicates that higher baseline values of PVR and age, and previous alpha-blocker use, predict an increase in the subscore and, therefore, a worsening of symptoms. For the placebo model, age, PSA, and previous alpha-blocker use were significant predictors of change in storage subscore (Table 3, Table S9).

Baseline IPSS storage subscore was a significant predictor of improvement in storage subscore for each of the active treatments (coefficient of –0.603; *p* < 0.001). Improvements in storage subscore were greater with CT than with either monotherapy across all baseline IPSS storage values; the improvement was greater with higher baseline storage subscores. Older age (coefficient of 0.019; *p* < 0.001), higher baseline PVR (coefficient of 0.001; *p* = 0.004), or previous alpha-blocker use (coefficient of 0.259; *p* = 0.001) predicted a significant worsening of storage symptoms.

While baseline PSA was not a significant predictor of change in storage subscore, the best model fitting the data is represented by the combination of the significant covariates at baseline and interaction between PSA and baseline storage subscore with treatments (Table S5). CT was predicted to improve IPSS storage subscores compared with tamsulosin across all baseline PSA values, with greater improvement with lower baseline PSA. The effect of dutasteride was similar to that of CT.

### IPSS voiding subscore

The predicted treatment benefit (reduction in voiding subscore) is greater with CT than with either monotherapy for most patient profiles within the CombAT inclusion criteria. The change from baseline in IPSS voiding subscore for an example patient profile in this model is shown in Fig. 1b.

The baseline covariates of Qmax, PVR, and baseline voiding subscore are significant predictors of the change in voiding subscore in the CombAT model (Table 3, Table S10). Overall, patient profiles with higher baseline voiding subscores benefit from each of the active treatments (coefficient of −0.652; *p* < 0.001), and improvement is greater with CT compared with dutasteride (coefficient of 0.085; *p* = 0.001) and tamsulosin (coefficient of 0.062; *p* = 0.012). Higher baseline PVR (coefficient of 0.003; *p* < 0.001) and lower Qmax (coefficient of −0.042; *p* < 0.001) at baseline predicted a significant worsening in voiding symptoms. For the placebo model, PSA and previous alpha-blocker use were significant predictors of change in voiding subscore (Table 3, Table S12).

Significant interaction effects were detected between baseline PV, PSA, and voiding subscores with treatment (Table 3). Patient profiles with lower PSA values were predicted to have significantly greater voiding symptom improvement with CT compared with either monotherapy. Higher baseline PSA values resulted in a comparable predicted treatment effect for dutasteride and CT.

### Nocturia (IPSS Q7)

The change from baseline in nocturia for an example patient profile is shown in Fig. 1c. The predicted treatment benefit for most patient profiles within the CombAT inclusion criteria was greater with CT compared with either monotherapy.

The baseline covariates of Qmax, PVR, nocturia severity, and age were significant predictors of the change in nocturia score in the CombAT model (Table 3, Table S14). Overall, patients with more severe nocturia at baseline benefited from all treatments (coefficient of –0.595; *p* < 0.001). Higher baseline PVR (coefficient of 0.001; *p* = 0.001) and lower baseline Qmax (coefficient of –0.008; *p* = 0.006) predicted a significant worsening in nocturia symptoms. For the placebo model, age, PSA, and baseline nocturia score were significant predictors of change in nocturia score (Table 3, Table S16).

A significant interaction between alpha-blocker use and treatment was observed. While previous alpha-blocker use predicted greater improvement in nocturia with CT or dutasteride, a significant worsening was predicted with tamsulosin (coefficient of 0.172; *p* = 0.009). Baseline nocturia score, age, and PVR also interacted significantly with treatment. Higher values of baseline nocturia score predicted greater improvements with CT compared with either monotherapy.

### QoL (IPSS Q8)

**Fig. 2:**
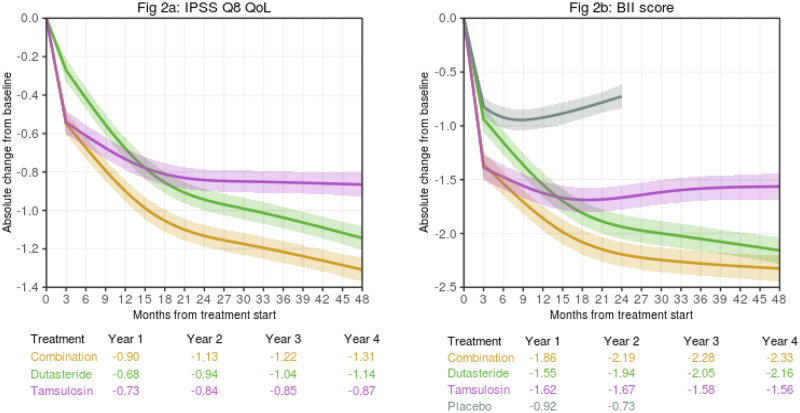
Examples of estimated trajectories over time of absolute change from baseline with 95% confidence intervals, by treatment group. **a** IPSS Q8 score, **b** BII score. **Obtained from** www.bphtool.com. Estimates for placebo are made using a model trained on monotherapies data only (2-year study duration). These examples consider mean values of covariates considered to estimate the trajectories for both treatments and placebo for the intended outcomes: (1) Common predictors for both outcomes: age, 66 years; PV, 55 mL; PVR, 68 mL; Qmax, 11 mL/s; PSA, 4 ng/mL; alpha-blocker use, no. (2) Additional outcome-specific predictors: IPSS QoL (Q8) score, 3; BII score, 5.

Figure 2a shows the change from baseline in QoL (IPSS Q8) for the example patient profile. Predictions for placebo were not possible since data for QoL (IPSS Q8) were not available in the dutasteride phase III studies. Once more, the predicted benefit for most profiles within CombAT inclusion criteria is greater with CT than with either monotherapy. The baseline covariates of total IPSS, IPSS Q8 score, Qmax, PVR, and previous alpha-blocker use were significant predictors of change in Q8 score (Table 3, Table S18).

Significant interactions were detected between baseline covariates of QoL (IPSS Q8) score, PV, age, Qmax, and treatment (Table 3). Higher scores at baseline were predictive of greater improvement in QoL (IPSS Q8) with CT compared with either monotherapy. For PV, the predicted difference of tamsulosin over CT was greater with higher baseline values.

### BII

The change from baseline in BII for an example patient profile is shown in Fig. 2b. CT provides greater predicted benefit compared with either monotherapy for most profiles within the CombAT inclusion criteria. Baseline age, PVR, BII score, and total IPSS were significant predictors of change in BII score (Table 3, Table S20). For the placebo model, total IPSS, PSA, and previous alpha-blocker use were significant predictors of change in BII score (Table 3, Table S22). A significant interaction between alpha-blocker use and treatment was observed (Table 3). Alpha-blocker use in the previous 12 months was predicted to provide greater benefit than no previous use for treatment with tamsulosin; however, both dutasteride and CT were predicted to provide greater benefit than tamsulosin alone.

## Discussion

We have previously developed multivariable predictive models using the largest available datasets of patients with LUTS/BPE at risk of disease progression to predict the change in IPSS total score and the risk of AUR and BPE-related surgery with different treatment regimens (placebo, dutasteride, tamsulosin, or CT) for up to 48 months, with baseline characteristics commonly used to define the risk of disease progression [13]. The current study extends this work by developing similar models to predict changes in IPSS storage subscore, IPSS voiding subscore, nocturia (IPSS Q7), QoL (IPSS Q8), and BII (bother). Consistent with results from post hoc analyses of average CombAT patient profiles [17, 18], in general, the predicted benefit over time of treatment with dutasteride/tamsulosin CT is greater than with either monotherapy for most patient risk profiles. Our results reinforce the benefit of a treatment approach that targets the underlying disease mechanism (5α-reductase inhibitors [5ARI]) in the short and long term in men with LUTS/BPE at risk of disease progression. They also provide insight on the impact of the studied treatments on different types of symptoms and QoL measures for individual patient profiles, which is typically not available from clinical trials (that address average treatment effects across a study population). In addition, the results allow greater understanding of the contribution of individual baseline characteristics that commonly define risk of LUTS/BPE disease progression.

Prior alpha-blocker use is a significant predictive factor of most outcomes. In addition, the models predict that the placebo effect is lower in men with a history of alpha-blocker use than in men without. This likely reflects differences in perceived benefit of active study treatment, which is likely to have been lower in men with alpha-blocker use in the previous 12 months than in men with no prior alpha-blocker use.

The predicted long-term effects of 5ARI-based treatment on storage symptoms and nocturia are also noteworthy. PVR is thought to be a multifactorial condition, with involvement of both bladder outlet obstruction (BOO) and bladder dysfunction (e.g., detrusor underactivity) [19]. Previous studies have suggested a role for 5ARIs in counteracting the molecular changes that lead to development and worsening of storage symptoms, probably as a result of PV reduction and improvement in BOO over time [20–23]. Several studies have also reported that 5ARI-based therapy can improve nocturia [18, 24]. Nocturia is associated with incomplete bladder emptying, OAB symptoms, and nocturnal polyuria, with the latter known to be highly prevalent in men with BPE [25].

While LUTS/BPE is rarely life-threatening, symptoms such as nocturia have been linked to increased mortality risk in men with the condition [26, 27]. The impact on QoL is also significant and should not be underestimated. This study allows visualization of the estimated change in total IPSS from baseline and corresponding QoL in individual profiles, to better understand the relevance of changes that ultimately will help guide treatment decisions [28].

As with our previous study [13], we found that each baseline covariate contributes differently to the predicted improvements. In addition, we identified significant interaction effects between covariates and treatment for each disease outcome, which should be interpreted in conjunction with the observed main effect of the covariate and treatment. This wealth of complexity poses a challenge in how to effectively present many and diverse results in an informative manner. For example, visualizing the effect of medical treatment in distinct patient profiles would require numerous nomograms or heatmaps. The educational interactive web-based tool (www.bphtool.com) was developed to facilitate visualization and understanding of the predicted outcomes for various combinations of baseline values within the eligibility criteria for the CombAT study [13]. The tool has been updated to enable visualization of change from baseline (absolute or percentage) in IPSS voiding and storage subscores, as well as nocturia score (IPSS Q7), QoL (IPSS Q8) score, and BII. It is, though, important to note that the tool is not intended on its own to substitute for medical advice nor to drive treatment decisions in real-world clinical practice. All such decisions should also consider a range of additional factors such as presence of comorbidities, risk of adverse events, and patient preferences and needs.

Potential limitations of our study should be acknowledged. The main limitation is that the modeling data and predictions may not be generalizable to the broader population of men with LUTS/BPE, as no information is provided for variables outside of the inclusion criteria used in the dutasteride source datasets (e.g., age <50 years, IPSS < 12, PV < 30 mL, PSA < 1.5 ng/mL). Also, placebo predictions were made using a model trained on 2-year monotherapy data only, since there was no placebo arm in CombAT; consequently, comparison of placebo with active therapies requires a strong assumption of exchangeability. In addition, it was not possible to validate model performance using data from trials with 2-year follow-up on active therapy, given the absence of such studies. Moreover, the models have not been assessed in the healthcare setting so are not, therefore, validated for clinical use. The models also did not evaluate adverse events, which are an important consideration when deciding on a personalized treatment approach. A further limitation is that the IPSS questionnaire does not allow assessment of incontinence, post-micturition symptoms, or bother due to different symptom types. Finally, due to the absence of suitable data from the source datasets, our models do not take account of other variables that might contribute to disease and treatment outcomes for men with LUTS/BPE (such as presence of comorbidities, intravesical prostatic protrusion, etc); this would be an interesting area of future research, along with extending the analyses to other available treatments and patient populations.

In conclusion, this predictive modeling study based on large datasets enhances our understanding of how risk factors for disease progression interact and affect the impact of treatment on different symptom types and QoL, reinforcing the importance of an individualized approach to LUTS/BPE management.

### Supplementary information

Supplementary information

Checklist

## Data Availability

Anonymized individual participant data and study documents from the studies mentioned within this paper can be requested for further research from www.clinicalstudydatarequest.com/Default.aspx. All the codes used to generate the outputs from this study are stored in a GSK-owned repository and can be requested for further research with permission from the owner.
