# Supplementary material for: Modeling study of the effect of placebo and medical therapy on storage and voiding symptoms, nocturia, and quality of life in men with prostate enlargement at risk for progression
Source: Prostate Cancer Prostatic Dis. 2023 Oct 4;27(3):469–77. doi: 10.1038/s41391-023-00731-w (PMC11319195; doi:10.1038/s41391-023-00731-w)
Supplement: Supplementary file 1 — Supplementary information [file 41391_2023_731_MOESM1_ESM.docx]

# Supplemental information

This file provides additional details on the Methods used in the study, and additional results on goodness of fit, cross-validation and predictive capability of each model for the different outcomes.

# Methods

## Main analytic approach

For each disease outcome explored (Table S1), an independent R-script file was provided, including the code for the statistical model, the validation process, and for extracting results such as tables and figures.

# Table S1. Outcome variables

| **Disease outcome** | **Collection timeframe** | **Definition** |
| --- | --- | --- |
| IPSS voiding sub-score – change from baseline | 24 months (phase III monotherapy source studies) or 48 months (CombAT study) | From the IPSS, it is the sub-score corresponding to the summatory of questions 1 (incomplete emptying), 3 (intermittency), 5 (weak stream), and 6 (straining to void). The values could range from 0 to 20. The change from baseline was obtained as the difference of the score at each point of time minus the baseline score |
| IPSS storage sub-score – change from baseline | 24 months (phase III monotherapy source studies) or 48 months (CombAT study) | From the IPSS, it is the sub-score corresponding to the summatory of questions 2 (frequency), 4 (urgency to void), and 7 (nocturia). The values could range from 0 to 15. The change from baseline was obtained as the difference of the score at each point of time minus the baseline score |
| IPSS Q7 nocturia score – change from baseline | 24 months (phase III monotherapy source studies) or 48 months (CombAT study) | From the IPSS, it is the nocturia sub-score related to question 7 with values between 0 to 5, higher scores are indication of worsening of health. The change from baseline was obtained as the difference of the score at each point of time minus the baseline score |
| IPSS Q8 QoL sub-score – change from baseline | Across 48 months (phase III monotherapy studies [24 months] did not evaluate Q8) | From the IPSS, it is the score related to question 8 with a range of values between 0 and 6, higher values being an indication of worsening of QoL. The change from baseline was obtained as the difference of the score at each point of time minus the baseline score |
| BII overall score – change from baseline | 24 months (phase III monotherapy source studies) or 48 months (CombAT study) | BII is summatory of four questions. The values could range from 0 to 13 with higher value being an indication of worsening of health. The change from baseline was obtained as the difference of the score at each point of time minus the baseline score |

BII, benign prostatic hyperplasia impact index; IPSS, International Prostate Symptom Score; QoL, quality of life.

# Table S2. Overview of studies selected for development and validation of the predictive models

| **Study number** | **Informed consent for data reuse** | **Study description** | **Dose and administration** | **Number of patients** | **Visits (months) and predictors availability** |
| --- | --- | --- | --- | --- | --- |
| ARIA3001  Dutasteride phase III monotherapy | Yes | A multicenter randomized double-blind, placebo-controlled, two-year parallel group study with a two-year open label phase | o.d. 0.5 mg dutasteride for 2 years  o.d. placebo for 2 years | Total: 1440  720 (50%)  720 (50%) | AUA-SI: 0, 1, 3, 6, 12, 18, 24  Prostate volume: 0, 1, 6, 12, 24  Qmax: 0, 1, 3, 6, 12, 18, 24  PSA: 0, 1, 3, 6, 12, 18, 24  Residual volume: 0, 1, 3, 6, 12, 18, 24  BII: 0, 1, 3, 6, 12, 18, 24  IPSS storage sub-score: 0, 1, 3, 6, 12, 18, 24  IPSS voiding sub-score: 0, 1, 3, 6, 12, 18, 24  IPSS Q7 nocturia sub-score: 0, 1, 3, 6, 12, 18, 24 |
| ARIA3002  Dutasteride phase III monotherapy | Yes | A multicenter randomized double-blind, placebo-controlled, two-year parallel group study with a two-year open label phase | o.d. 0.5 mg dutasteride for 2 years  o.d. placebo for 2 years | Total: 1362  677 (49.7%)  685 (50.29%) | AUA-SI: 0, 1, 3, 6, 12, 18, 24  Prostate volume: 0, 1, 6, 12, 24  Qmax: 0, 1, 3, 6, 12, 18, 24  PSA: 0, 1, 3, 6, 12, 18, 24  Residual volume: 0, 1, 3, 6, 12, 18, 24  BII: 0, 1, 3, 6, 12, 18, 24  IPSS storage sub-score: 0, 1, 3, 6, 12, 18, 24  IPSS voiding sub-score: 0, 1, 3, 6, 12, 18, 24  IPSS Q7 nocturia sub-score: 0, 1, 3, 6, 12, 18, 24 |
| ARIB3003  Dutasteride phase III monotherapy | Yes | A multicenter randomized double-blind, placebo-controlled, two-year parallel group study with a two-year open label phase | o.d. 0.5 mg dutasteride for 2 years  o.d. placebo for 2 years | Total: 1523  770 (50.56%)  753 (49.44%) | AUA-SI: 0, 1, 3, 6, 12, 18, 24  Prostate volume: 0, 1, 6, 12, 24  Qmax: 0, 1, 3, 6, 12, 18, 24  PSA: 0, 1, 3, 6, 12, 18, 24  Residual volume: 0, 1, 3, 6, 12, 18, 24  BII: 0, 1, 3, 6, 12, 18, 24  IPSS storage sub-score: 0, 1, 3, 6, 12, 18, 24  IPSS voiding sub-score: 0, 1, 3, 6, 12, 18, 24  IPSS Q7 nocturia sub-score: 0, 1, 3, 6, 12, 18, 24 |
| ARI40005  CombAT | Yes | A multicenter, randomized, double-blind, four-year parallel-group study. | o.d. 0.4 mg tamsulosin for 4 years  o.d. 0.5 mg dutasteride for 4 years  o.d. 0.5 mg dutasteride and 0.4 mg tamsulosin therapy for 4 years | Total: 4841  1609 (33.25%)  1623 (33.52%)  1609 (33.23%) | IPSS: 0, 3, 6, 9, 12, 15, 18, 21, 24, 27, 30, 33, 36, 39, 42, 45, 48  Prostate volume: 0, 12, 24, 36, 48  Qmax: 0, 6, 12, 18, 24, 30, 36, 42 and 48 PSA: 0, 12, 24, 36, 48  Residual volume: 0, 6, 12, 18, 24, 30, 36, 42, 48  BII: 0, 1, 3, 6, 12, 18, 24, 27, 30, 33, 36, 39, 42, 45, 48  IPSS storage sub-score: 0, 1, 3, 6, 12, 18, 24, 27, 30, 33, 36, 39, 42, 45, 48  IPSS voiding sub-score: 0, 1, 3, 6, 12, 18, 24, 27, 30, 33, 36, 39, 42, 45, 48  IPSS Q7 nocturia sub-score: 0, 1, 3, 6, 12, 18, 24, 27, 30, 33, 36, 39, 42, 45, 48  IPSS Q8 QoL score: 0, 1, 3, 6, 12, 18, 24, 27, 30, 33, 36, 39, 42, 45, 48 |

All treatments were given as a once-daily dosing regimen. The AUA-SI is the symptoms index score alone. The AUA-SI with the IPSS Q8 QoL equals the IPSS.

For the monotherapy studies (ARIA/B) the IPSS score is synonymous with the AUA-SI score.
AUA-SI, American Urology Association Symptom Index; BII, benign prostatic hyperplasia impact index; IPSS, International Prostate Symptom Score; o.d., once daily; PSA, prostate-specific antigen; Qmax, maximum urine flow rate; QoL, quality of life.

## Modelling strategy

In the case of longitudinal change from baseline in each of the disease outcome explored, the serial measurements taken over time implies within-subject dependency. To be consistent with the previous study, where the change from baseline of the total International Prostate Symptom Score (IPSS) score was predicted, the prediction of the change from baseline has been chosen as the response variable (i.e., the difference of the value of the sub-score at a point in time minus the storage sub-score at time 0 [baseline]). To draw appropriate inference, this correlation must be accounted for in the analysis. In the original study [1], the correlation is a nuisance parameter and is of no specific interest in generating predicted values for future subjects. This outcome is analyzed by the generalized least squares (GLS) approach, which is a natural extension of a simple linear model that relies on ordinary least squares. GLS allows us to include a correlation structure matrix to take into account the longitudinal aspect of the outcome. The GLS works by assuming that, conditional on the predictors, the outcome (i.e., change from baseline) follows a multivariate normal distribution. The GLS can then incorporate ‘time’ as an additional predictor. Here, we allow this to be flexibly modelled and use a non-linear transformation in the form of a restricted cubic spline, and further assume that the shape of the predictor-outcome association with time also depends on the treatment [2].

Acknowledging the heterogeneity of treatment effect due to unequal baseline covariate, we perform a manual backward elimination procedure to choose whether a model comprising of interaction between treatment and other baseline variables is significant. The statistical test associated with the interaction parameters, along with the Akaike’s Information Criteria (AIC), Bayesian Information Criteria (BIC), and likelihood metrics, are used to compare two nested models: one model with no interaction arm and a second model containing the interaction between the treatment and the baseline covariate. Furthermore, a manual forward selection procedure for checking the relevance of interactions between covariates and the baseline response is performed in order to test for heterogeneity of baseline responses due to unequal covariate values.

Finally, predictors used are aligned with the previous study [1] while also taking into account the particularities of this new analysis (Table 2, main manuscript). Goodness of fit for both models are extracted and the most adequate and robust model is used for final predictions.

The predictive model is evaluated for performance using different metrics for variable selection and model evaluation. We evaluate variable selection using unbiased estimates of the model prediction error via the AIC and BIC metrics. Evaluation of the final GLS models is provided via the root mean squared error (RMSE) metric as a representation of the differences between values predicted by a model and the observed values (i.e.,an estimator of the standard deviation of the residuals). We obtain the evaluation metrics from a 10-fold cross-validation process, where subjects are divided randomly into 10 parts; we then use nine of those parts for training and one for testing, repeating the procedure 10 times (each time reserving as test data one of the ten parts). In Figure S1, an example for a 5-fold cross-validation processed is shown; the process for a 10-fold cross-validation follows the same logic but with 10 folds instead of 5.

# Figure S1. 5-fold CV logic


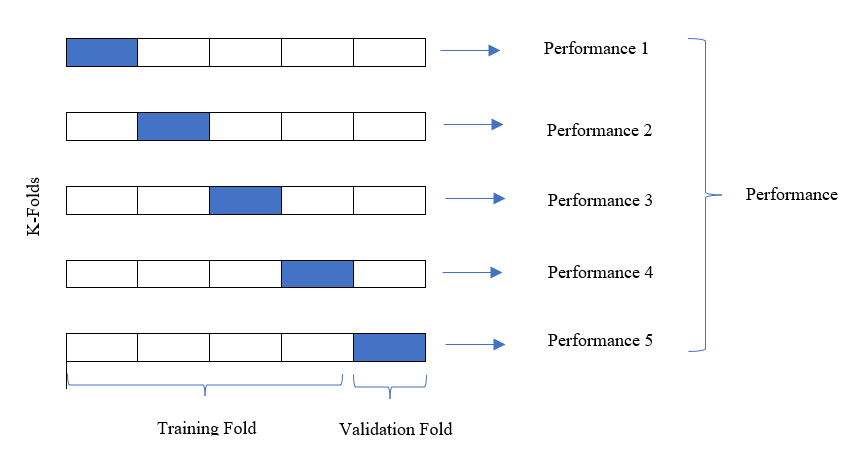


CV, cross-validation

Missing data were not imputed, but a 'last observation carried forward’ approach was used (similar to that used to develop the initial predictive analytic solution [1]). Overfitting was adjusted using the ‘uniform shrinkage’ method [3].

## Handling missing data

## Endpoints

Attrition (loss of study units from a sample) is typical in clinical trials. For ARI40005, ARIA3001, ARIA3002, and ARIB3003, patients not completing the study have been previously reported as ranging between 30–39% [4,5]. For the case of longitudinal IPSS prediction, we note that the original trials pre-dated the more recent International Council for Harmonisation of Technical Requirements for Registration of Pharmaceuticals for Human Use Topic E 9 Statistical Principles for Clinical Trials (ICH E9) addendum on estimands and that the approach adopted at that time uses last observation carried forward (LOCF) to handle any drop-out or loss-to-follow-up [6]. In the present analysis, we adopt the same approach (LOCF), thus enabling a broadly similar interpretation of the estimands utilized both in the original source studies and in the risk modelling (note: recognizing that the analyses represent different summary measures to the original [1] CombAT and phase III monotherapy source studies).

## Baseline characteristics

The loss of information across baseline characteristics posed a problem in the analysis. One way to handle this is to perform a complete case analysis, whereby patients with the missing baseline values are removed. This is particularly wasteful as the collected outcome data and their contribution in the estimation of the effects are lost. Complete case analyses therefore suffer from selection bias and loss of information [7].

A proportion of missing data are present across the baseline characteristics in the benign prostatic hyperplasia trials data. We implemented an imputation strategy that relies on a missing-at-random assumption. The assumption is that entry of given variables may be missing as a direct result of other measured (and thus observed) variables, in which case, a dependency exists between the probability of *missingness* and some (or all) of the observed covariates. We implement a multiple imputation strategy to include all the candidate predictors and cover imputation of missing data for model development. Baseline variables missing in the validation datasets are imputed in a conditional fashion, using only the data that was left aside for validation (i.e., not used to build the model).

As is typical for clinical trials, we expect the overall extent of missingness across the baseline characteristics to be low [7]. As such, it is likely that a complete case analysis, or indeed a simple single imputation strategy, would yield similar results. However, it is well regarded in the development of prediction models that multiple imputation of missing data is a good methodological practice and so the above-described approach was implemented throughout [8].

## Overfitting

Overfitting occurs when a model is trained to recognize the idiosyncrasies of the *development* data and as a result generalizes poorly to unseen validation data (i.e., test data). In practice, this typically results in the estimation of outcome-predictor relationships that are biased towards being larger (and thus predicted responses that are too big). Consequently, it was desirable to impose some form of shrinkage on the effects to mitigate for this [7]. A simple solution, known as *uniform shrinkage*, aims to estimate a shrinkage factor that is less than one. All the model coefficients can thus be reduced (as a multiple) by this shrinkage factor in a bid to help generalizability.

An empirical approach can be followed to estimate this shrinkage factor for any given model as follows [3]:

1. Generate a bootstrap sample (with replacement) from the validation test data of the same size as the training data sample;
2. Fit a model to this bootstrap sample and estimate outcome-predictor effects;
3. Use this bootstrap model to derive a linear predictor score for each patient in the original sample;
4. Estimate the slope of this linear predictor and compare it with the corresponding value from the training data.

Repeating the above steps many times and averaging the results provides an empirical estimate for the uniform shrinkage. The observed value of the shrinkage factor was between 0.998 and 1.005 and therefore we can conclude that there was no significant overfitting in the model.

## Software

Analyses were conducted using R version 4.2.2 in a secure and validated environment.

# Results

## Descriptive analysis of baseline variables

# Table S3. Mean values and $\boldsymbol{\chi}^{\mathbf{2}}$ tests between combination, dutasteride, and tamsulosin groups for covariates used in the model from ARI40005

| **Variable** | **Levels** | **Combination** | **Dutasteride** | **Tamsulosin** | **P-value** |
| --- | --- | --- | --- | --- | --- |
| Age at treatment start (year) | Mean (SD) | 66.0 (7.0) | 66.0 (7.9) | 66.2 (7.0) | 0.653 |
|  | Median (IQR^†^) | 66.0 (61–71) | 66.0  (61.0–71.0) | 66.0  (61.0–71.0) | 0.524 |
| Baseline prostate volume (mL) | Mean (SD) | 54.7 (23.5) | 54.6 (23) | 55.8 (24.2) | 0.271 |
|  | Median (IQR^†^) | 48.9  (39.2–63.2) | 48.4  (38.5–63.2) | 49.6  (38.7–65) | 0.324 |
| Baseline prostate volume (mL) | Mean (SD) | 68.2 (66.1) | 67.4 (63.5) | 67.7 (65.2) | 0.939 |
|  | Median (IQR^†^) | 50 (20–96) | 50 (20–100) | 50  (20–100) | 0.953 |
| Baseline Qmax (mL/s) with minimum voiding volume > 125 mL | Mean (SD) | 10.9 (3.6) | 10.6 (3.6) | 10.6 (3.6) | 0.074 |
|  | Median (IQR^†^) | 10.6 (8.4–12.8) | 10.3 (8.0–12.7) | 10.3 (8.0–12.6) | 0.036 |
| Baseline PSA (ng/mL) | Mean (SD) | 4.0 (2.1) | 3.92 (2.1) | 4.0 (2.1) | 0.221 |
|  | Median (IQR^†^) | 3.4 (2.4–5.1) | 3.4 (2.3–5.1) | 3.5 (2.4–5.2) | 0.139 |
| Baseline total IPSS | Mean (SD) | 16.6 (6.3) | 16.4 (6.0) | 16.4 (6.1) | 0.637 |
|  | Median (IQR^†^) | 16 (12–21) | 16 (12–20) | 16 (12–20) | 0.678 |
| Baseline IPSS storage sub-score | Mean (SD) | 7.3 (3.0) | 7.2 (2.9) | 7.2 (2.9) | 0.628 |
|  | Median (IQR^†^) | 7.0 (5.0–9.0) | 7.0 (5.0–9.0) | 7.0 (5.0–9.0) | 0.817 |
| Baseline IPSS voiding sub-score | Mean (SD) | 9.3 (4.3) | 9.2 (4.3) | 9.2 (4.2) | 0.732 |
|  | Median (IQR^†^) | 9.0 (6.0–12.0) | 9.0 (6.0–12.0) | 9.0 (6.0–12.0) | 0.777 |
| Baseline nocturia score | Mean (SD) | 2.4 (1.2) | 2.4 (1.2) | 2.4 (1.2) | 0.817 |
|  | Median (IQR^†^) | 2 (2–3) | 2 (2–3) | 2 (2–3) | 0.881 |
| Baseline BPH Impact Index Score | Mean (SD) | 5.3 (3.1) | 5.3 (3.0) | 5.3 (3.1) | 0.898 |
|  | Median (IQR^†^) | 5.0 (3.0–8.0) | 5.0 (3.0–8.0) | 5.0 (3.0–7.0) | 0.753 |
| Baseline IPSS Q8 score | Mean (SD) | 3.3 (0.9) | 3.3 (0.9) | 3.3 (0.9) | 0.977 |
|  | Median (IQR^†^) | 4 (3–5) | 4 (3–5) | 4 (3–5) | 0.950 |
| AB | N (%^‡^) | 1264 (78.7) | 1261 (77.7) | 1266 (78.7) | 0.761 |
|  | Y (%^‡^) | 343 (21.3) | 361 (22.3) | 343 (21.3) |  |

P-values for the mean are obtained from the ANOVA test, p-values for median are obtained using the non-parametric Kruskal test and the p-value for AB has been obtained using the Chi-square test.
^†^IQR stands for the interval of the IQR (25–75%).
^‡^As AB is a categorical variable, instead of the mean or the median, the % of observations in each category is presented.

AB, alpha-blocker usage (yes/no) in the last 12 months; ANOVA, analysis of variance; BPH, benign prostatic hyperplasia; IPSS, International Prostate Symptom Score; IQR, interquartile range; PSA, prostate-specific antigen; Qmax, maximum urinary flow rate; SD, standard deviation.

# Table S4. Mean values and t-tests between dutasteride and placebo groups for covariates used in the model from dutasteride phase III monotherapy studies

| **Variable** | **Levels** | **Dutasteride** | **Placebo** | **P-value** |
| --- | --- | --- | --- | --- |
| Age at treatment start (year) | Mean (SD) | 66.5 (7.6) | 66.1 (7.4) | 0.046 |
|  | Median (IQR) | 67 (61–72) | 66 (61–71) | 0.063 |
| Baseline prostate volume (mL) | Mean (SD) | 54.9 (23.9) | 54.0 (21.9) | 0.165 |
|  | Median (IQR) | 48.7 (38.5–63.1) | 48.3 (39–62.3) | 0.491 |
| Baseline postvoid residual volume (mL) | Mean (SD) | 76.6 (75.0) | 74.9 (69.6) | 0.441 |
|  | Median (IQR) | 56.0 (20–112) | 59.0 (20–109) | 0.864 |
| Baseline Qmax (mL/s) with minimum voiding volume > 125 mL | Mean (SD) | 10.1 (3.5) | 10.3 (3.6) | 0.036 |
|  | Median (IQR) | 9.9 (7.6–12.2) | 10.2 (7.9–12.5) | 0.031 |
| Baseline PSA (ng/mL) | Mean (SD) | 4.0 (2.1) | 4.0 (2.1) | 0.668 |
|  | Median (IQR) | 3.4 (2.3–5.3) | 3.5 (2.3–5.2) | 0.936 |
| Baseline total IPSS | Mean (SD) | 17.1 (6.0) | 17.2 (6.1) | 0.606 |
|  | Median (IQR) | 17 (13–21) | 17 (13–21) | 0.886 |
| Baseline IPSS storage subscore | Mean (SD) | 7.7 (3.0) | 7.8 (3.0) | 0.354 |
|  | Median (IQR) | 8.0 (5.0–10.0) | 8.0 (6.0–10.0) | 0.421 |
| Baseline IPSS voiding subscore | Mean (SD) | 9.4 (4.1) | 9.4 (4.3) | 0.980 |
|  | Median (IQR) | 9.0 (7.0–12.0) | 9.0 (6.0–12.0) | 0.654 |
| Baseline BPH Impact Index Score | Mean (SD) | 4.1 (2.7) | 4.0 (2.8) | 0.368 |
|  | Median (IQR) | 4.0 (2.0–6.0) | 4.0 (2.0–6.0) | 0.255 |
| Baseline IPSS Q7 nocturia | Mean (SD) | 2.4 (1.2) | 2.4 (1.2) | 0.621 |
|  | Median (IQR) | 2 (2–3) | 2 (2–3) | 0.779 |
| AB | N (%) | 1815 (83.8) | 1822 (84.4) | 0.573 |
|  | Y (%) | 352 (16.2) | 336 (15.6) |  |

IPSS Q8 data was not included for placebo studies.
P-values correspond to the ANOVA test for age and QMAX, Kruskal Wallis test for PV and PSA, and Chi-square test for AB.
AB, alpha-blocker usage (yes/no) in the last 12 months; ANOVA, analysis of variance; BPH, benign prostatic hyperplasia; IPSS, International Prostate Symptom Score; IQR, interquartile range; PSA, prostate-specific antigen; PV, prostate volume; Qmax, maximum urinary flow rate; SD, standard deviation.

Table S5 shows the AIC, BIC, and RMSE values from the GLS model without any interaction and the model obtained using the significant interactions among the baseline covariates and treatment and the baseline covariates and baseline storage sub-score.

The AIC and BIC are very similar and the RMSE is the same (2.573). For the rest of the analysis, the model with interactions has been used for better understanding of the covariate’s effects. The best model fitting the data is represented by the combination of the covariates at baseline and the interactions between prostate-specific antigen (PSA) and baseline storage score with treatments, with a resulting AIC of 247199.4 and BIC of 247424.6.

# Table S5. Goodness of fit measure

| Outcome | Model | AIC | BIC | RMSE |
| --- | --- | --- | --- | --- |
| IPSS storage subscore | CombAT |  |  |  |
|  | Without interaction terms | 247180.5 | 247369.7 | 2.573 |
|  | With interaction terms | 247199.4 | 247424.6 | 2.573 |
|  | Placebo |  |  |  |
|  | Without interaction terms | 95501.39 | 95636.95 | 2.611 |
|  | With interaction terms | 95516.38 | 95683.83 | 2.605 |
| IPSS voiding subscore | CombAT |  |  |  |
|  | Without interaction terms | 284298.5 | 284487.5 | 3.596 |
|  | With interaction terms | 284330.5 | 284591.7 | 3.585 |
|  | Placebo |  |  |  |
|  | Without interaction terms | 108665.6 | 108801.2 | 3.603 |
|  | With interaction terms | 108687.2 | 108854.6 | 3.595 |
| Nocturia | CombAT |  |  |  |
|  | Without interaction terms | 145824.0 | 146013.2 | 1.012 |
|  | With interaction terms | 145858.6 | 146110.9 | 1.010 |
|  | Placebo |  |  |  |
|  | Without interaction terms | 59732.1 | 59883.6 | 1.072 |
|  | With interaction terms | 59725.1 | 59860.7 | 1.073 |
| IPSS Q8 | CombAT |  |  |  |
|  | Without interaction terms | 152916.5 | 153114.8 | 1.176 |
|  | With interaction terms | 152982.0 | 153261.4 | 1.173 |
| BII | CombAT |  |  |  |
|  | Without interaction terms | 235772.6 | 235971.0 | 2.374 |
|  | With interaction terms | 235783.7 | 236018.1 | 2.372 |
|  | Placebo |  |  |  |
|  | Without interaction terms | 86063.64 | 86206.78 | 2.196 |
|  | With interaction terms | 86076.82 | 86243.82 | 2.190 |

AIC, Akaike’s Information Criteria; BIC, Bayesian Information Criteria; BII, benign prostatic hyperplasia impact index; IPSS, International Prostate Symptom Score; RMSE, root mean squared error.

## IPSS storage sub-score

## CombAT model

Table S6 shows the estimated coefficients, standard errors, and p-values. The baseline covariates post-void residual urine (PVR), alpha-blocker (AB), age, and baseline storage sub-score are significant predictors of the change from baseline IPSS storage sub-score. The positive sign of the coefficient indicates that higher values of PVR and age at baseline or being a patient with previous history of AB use (AB = Yes) predicts an increase from baseline and therefore a significant worsening on the symptoms. In the case of baseline storage sub-score symptoms, higher baseline values leads to a decrease from baseline and therefore an improvement on the symptoms. Storage baseline values interact with treatment indicating that depending on the treatment and the baseline storage values, the impact on the prediction changes. Overall, patients with higher baseline storage subscore symptoms benefit from all treatments (coefficient of -0.603; p-value <0.001). However, patients with higher baseline value of IPSS storage subscore in the combined treatment arm experience greater improvement in symptom score compared with similar patients in the dutasteride arm, since the coefficient corresponding to the interaction of the dutasteride with baseline storage sub-score is 0.053 with a p-value of 0.038.

# Table S6. Estimated parameters, standard errors, and p-values for the best fitting CombAT model storage subscore

| Summary coefficients | | | | |
| --- | --- | --- | --- | --- |
|  | Estimate | Std error | t-statistic | p-value |
| Intercept | 1.433 | 0.348 | 4.120 | <0.001*** |
| Baseline postvoid residual volume | 0.001 | 0.001 | 2.910 | 0.004** |
| AB=Y | 0.259 | 0.074 | 3.500 | 0.001** |
| Age at treatment start | 0.019 | 0.004 | 4.340 | <0.001*** |
| Baseline Qmax | -0.010 | 0.008 | -1.160 | 0.246 |
| Baseline prostate volume | 0.001 | 0.001 | 1.080 | 0.281 |
| Baseline PSA | 0.007 | 0.026 | 0.290 | 0.775 |
| Baseline IPSS storage sub-score | -0.603 | 0.018 | -33.810 | <0.001*** |
| TMTDG=Dutasteride | 0.508 | 0.251 | 2.030 | 0.043* |
| TMTDG=Tamsulosin | -0.315 | 0.251 | -1.250 | 0.211 |
| MONTH | -0.059 | 0.005 | -11.740 | <0.001*** |
| MONTH’ | 0.100 | 0.020 | 5.040 | <0.001*** |
| MONTH’’ | -0.169 | 0.046 | -3.650 | <0.001*** |
| B_PSA*TMTDG=Dutasteride | -0.067 | 0.036 | -1.860 | 0.062 |
| B_PSA*TMTDG=Tamsulosin | 0.008 | 0.036 | 0.230 | 0.817 |
| B_storage_sub_score*TMTDG=Dutasteride | 0.053 | 0.026 | 2.080 | 0.038* |
| B_storage_sub_score*TMTDG=Tamsulosin | 0.029 | 0.025 | 1.140 | 0.256 |
| TMTDG=Dutasteride*MONTH | -0.015 | 0.007 | -2.060 | 0.040* |
| TMTDG=Tamsulosin*MONTH | 0.032 | 0.007 | 4.490 | <0.001*** |
| TMTDG=Dutasteride*MONTH’ | 0.044 | 0.028 | -0.900 | 0.115 |
| TMTDG=Tamsulosin*MONTH’ | -0.025 | 0.066 | 0.530 | 0.367 |
| TMTDG=Dutasteride*MONTH’’ | -0.098 | 0.065 | -1.510 | 0.132 |
| TMTDG=Tamsulosin*MONTH’’ | 0.035 | 0.066 | 0.530 | 0.596 |

P-values have not been adjusted for multiplicity.

Interactions with associated p-values higher than 0.05 and insignificant improvement of the model have been excluded.

*p-value between 0.01 and 0.05; **p-value between 0.001 and 0.01; ***p-value smaller than 0.001.

AB, alpha-blocker usage (yes/no) in the last 12 months; IPSS, International Prostate Symptom Score; PSA, prostate-specific antigen; Qmax, maximum urinary flow rate; std error, standard error.

## Cross-validation and predictive capability

Table S7 shows the RMSE from the 10-fold cross validation process. The results from the train and test data for each partition of the data are provided. It is observed that RMSE results for the test data sets are like the results obtained from the training model, indicating that the models are as good for predictions of new data as for the training set.

# Table S7. RMSE values from the 10-fold cross validation process. CombAT model storage subscore

| Iteration CV | RMSE (train) | RMSE (test) |
| --- | --- | --- |
| 1 | 2.570 | 2.554 |
| 2 | 2.565 | 2.584 |
| 3 | 2.568 | 2.561 |
| 4 | 2.569 | 2.564 |
| 5 | 2.564 | 2.605 |
| 6 | 2.579 | 2.483 |
| 7 | 2.570 | 2.562 |
| 8 | 2.564 | 2.606 |
| 9 | 2.568 | 2.566 |
| 10 | 2.558 | 2.647 |

CV, cross-validation; RMSE, root mean squared error.

## Placebo model

Table S5 shows the AIC, BIC, and RMSE values from the GLS model without any interaction, as well as for the model obtained using the significant interactions among the baseline covariates and treatment, the baseline covariates, and baseline storage sub-score. Both models present very similar results for all the goodness of fit measures analyzed. The model with interactions has been used for the rest of the analysis for a better understanding of the interactions between the covariates.

Table S8 shows the estimated parameters, standard errors, and p-values. The baseline covariates PSA, AB, and age are significant predictors of the storage change score. The interactions between age and prostate volume (PV) with treatment were significant and improve the goodness of fit of the model. Two further interactions between age and PVR with baseline storage score significantly improved the predicted model.

# Table S8. Estimates from the fitted model to the change from baseline on the storage sub-score using the placebo and dutasteride studies. Placebo model storage subscore

| Summary coefficients | | | | |
| --- | --- | --- | --- | --- |
|  | Value | Std error | t-statistic | p-value |
| Intercept | 0.805 | 0.837 | 0.962 | 0.336 |
| Baseline PSA | -0.052 | 0.016 | -3.348 | 0.001** |
| Baseline Qmax | -0.004 | 0.009 | -0.503 | 0.615 |
| Baseline PVR | -0.001 | 0.001 | -0.744 | 0.457 |
| AB=Y | 0.489 | 0.085 | 5.751 | <0.001*** |
| Age at treatment start | 0.033 | 0.012 | 2.651 | 0.008** |
| Baseline IPSS storage sub-score | -0.183 | 0.095 | -1.935 | 0.053 |
| Baseline prostate volume | -0.001 | 0.002 | -0.504 | 0.614 |
| TMTDG=Placebo | 0.426 | 0.565 | 0.753 | 0.452 |
| MONTH | -0.189 | 0.020 | -9.295 | <0.001*** |
| MONTH’ | 1.369 | 0.272 | 5.039 | <0.001*** |
| MONTH’’ | -1.668 | 0.351 | -4.751 | <0.001*** |
| AGE*B_storage_sub_score | -0.004 | 0.001 | -2.521 | 0.012* |
| B_PVR*B_storage_sub_score | 0.000 | 0.000 | 2.105 | 0.035* |
| AGE*TMTDG=Placebo | -0.014 | 0.008 | -1.671 | 0.095 |
| B_PV*TMTDG=Placebo | 0.010 | 0.003 | 3.543 | <0.001*** |
| TMTDG=Placebo*MONTH | 0.033 | 0.029 | 1.147 | 0.251 |
| TMTDG=Placebo*MONTH’ | 0.192 | 0.384 | 0.499 | 0.618 |
| TMTDG=Placebo*MONTH’’ | -0.271 | 0.497 | -0.547 | 0.585 |

P-values have not been adjusted for multiplicity.

Interactions with associated p-values higher than 0.05 and insignificant improvement of the model have been excluded.

*p-value between 0.01 and 0.05; **p-value between 0.001 and 0.01; ***p-value smaller than 0.001.
AB, alpha-blocker usage (yes/no) in the last 12 months; IPSS, International Prostate Symptom Score; PSA, prostate-specific antigen; PV, prostate volume; PVR, post-void residual urine; Qmax, maximum urinary flow rate; std error, standard error.

## Cross-validation and predictive capability

# Table S9. RMSE values from the 10-fold cross validation process. Placebo model storage sub-score

| Iteration number | RMSE  (train) | RMSE  (test) |
| --- | --- | --- |
| 1 | 2.608 | 2.555 |
| 2 | 2.588 | 2.746 |
| 3 | 2.618 | 2.454 |
| 4 | 2.594 | 2.681 |
| 5 | 2.603 | 2.618 |
| 6 | 2.599 | 2.639 |
| 7 | 2.6 | 2.631 |
| 8 | 2.617 | 2.476 |
| 9 | 2.6 | 2.625 |
| 10 | 2.597 | 2.659 |

RMSE, root mean squared error.

## IPSS voiding subscore

## CombAT model

Table S5 shows the AIC, BIC, and RMSE values from the GLS model without any interaction, as well as for the model obtained using the significant interactions among the baseline covariates and treatment and the baseline covariates and baseline voiding subscore. Both models present very similar results for all the goodness of fit measures analyzed. The model with interactions has been used for the rest of the analysis for better understanding of the interactions between the covariates.

Table S10 shows a summary of the results from the final model. The estimated coefficients associated with each predictor and the relevant interactions are provided. Baseline Qmax, PVR, and voiding sub-score values are significantly associated with change. Patients with higher Qmax or voiding sub-score values at baseline predict a better improvement over time. On the other hand, patients with higher PVR at baseline predict less improvement over time. Significant interactions effects between PV, PSA, and baseline voiding scores with treatment are detected.

# Table S10. Estimates from the fitted model. CombAT model voiding subscore

| Summary coefficients | | | | |
| --- | --- | --- | --- | --- |
|  | Estimate | Std error | t-statistic | p-value |
| Intercept | 3.880 | 0.550 | 7.060 | <0.001*** |
| Baseline PVR | 0.003 | 0.001 | 4.090 | <0.001*** |
| AB=Y | -0.166 | 0.250 | -0.660 | 0.507 |
| Age at treatment start | -0.006 | 0.006 | -0.990 | 0.321 |
| Baseline Qmax | -0.042 | 0.012 | -3.500 | 0.001** |
| Baseline prostate volume | -0.003 | 0.005 | -0.530 | 0.598 |
| Baseline IPSS voiding sub-score | -0.652 | 0.028 | -23.270 | <0.001*** |
| Baseline PSA | 0.075 | 0.038 | 1.970 | 0.049* |
| TMTDG=Dutasteride | 0.942 | 0.391 | 2.410 | 0.016* |
| TMTDG=Tamsulosin | -1.044 | 0.384 | -2.720 | 0.007** |
| MONTH | -0.084 | 0.007 | -12.170 | <0.001*** |
| MONTH’ | 0.134 | 0.027 | 4.920 | <0.001*** |
| MONTH’’ | -0.210 | 0.063 | -3.310 | 0.001** |
| B_PV*B_voiding_sub_score | -0.001 | 0.000 | -1.940 | 0.053 |
| AB=Y*B_voiding_sub_score | 0.056 | 0.024 | 2.390 | 0.017* |
| B_voiding_sub_score*TMTDG=Dutasteride | 0.085 | 0.025 | 3.440 | 0.001** |
| B_voiding_sub_score*TMTDG=Tamsulosin | 0.062 | 0.025 | 2.520 | 0.012* |
| B_PV*TMTDG=Dutasteride | 0.003 | 0.005 | 0.540 | 0.586 |
| B_PV*TMTDG=Tamsulosin | 0.017 | 0.005 | 3.550 | <0.001*** |
| B_PSA*TMTDG=Dutasteride | -0.121 | 0.054 | -2.250 | 0.024* |
| B_PSA*TMTDG=Tamsulosin | -0.109 | 0.054 | -2.030 | 0.043* |
| TMTDG=Dutasteride*MONTH | -0.045 | 0.010 | -4.660 | <0.001*** |
| TMTDG=Tamsulosin*MONTH | 0.066 | 0.010 | 6.740 | <0.001*** |
| TMTDG=Dutasteride*MONTH’ | 0.125 | 0.038 | 3.250 | 0.001** |
| TMTDG=Tamsulosin*MONTH’ | -0.075 | 0.039 | -1.950 | 0.051 |
| TMTDG=Dutasteride*MONTH’’ | -0.280 | 0.089 | -3.130 | 0.002** |
| TMTDG=Tamsulosin*MONTH’’ | 0.102 | 0.090 | 1.130 | 0.259 |

P-values have not been adjusted for multiplicity.

Interactions with associated p-values higher than 0.05 and insignificant improvement of the model have been excluded.

*p-value between 0.01 and 0.05; **p-value between 0.001 and 0.01; ***p-value smaller than 0.001.
AB, alpha-blocker usage (yes/no) in the last 12 months; IPSS, International Prostate Symptom Score; PSA, prostate-specific antigen; PV, prostate volume; PVR, post-void residual urine; Qmax, maximum urinary flow rate; std error, standard error.

## Cross-validation and predictive capability

# Table S11. RMSE values from the 10-fold cross validation process. CombAT model voiding sub-score

| Iteration number | RMSE  (train) | RMSE  (test) |
| --- | --- | --- |
| 1 | 3.576 | 3.672 |
| 2 | 3.587 | 3.562 |
| 3 | 3.591 | 3.537 |
| 4 | 3.580 | 3.648 |
| 5 | 3.597 | 3.488 |
| 6 | 3.579 | 3.658 |
| 7 | 3.589 | 3.559 |
| 8 | 3.588 | 3.552 |
| 9 | 3.585 | 3.587 |
| 10 | 3.573 | 3.693 |

RMSE, root mean squared error.

## Placebo model

Table S5 shows the AIC, BIC, and RMSE values from the GLS model without any interaction, as well as for the model obtained using the significant interactions among the baseline covariates and treatment, the baseline covariates, and baseline voiding sub-score. Both models present very similar results for all the goodness of fit measures analyzed. The model with interactions has been used for the rest of the analysis for better understanding of the interactions between the covariates.

Table S12 shows the results from the final fitted model to the change of voiding scores for the studies of placebo and dutasteride. The baseline covariates PSA and AB are significant predictor of the voiding change score.

The positive sign of the coefficient for AB indicates that being a patient on AB previously predicts higher values of the change and therefore a significant worsening of symptoms. In the case of PSA, the opposite conclusion can be reached, higher values lead to lower values of the change and therefore an improvement on the symptoms.

An interaction between PV and treatment was significant. Also, there were interaction effects between voiding baseline scores with age, Qmax, and PVR.

# Table S12. Estimates from the fitted model. Placebo model voiding sub-score

| Summary coefficients | | | | |
| --- | --- | --- | --- | --- |
|  | Estimate | Std error | t-statistic | p-value |
| Intercept | 2.307 | 1.035 | 2.230 | 0.026* |
| Baseline PSA | -0.051 | 0.022 | -2.359 | 0.018* |
| AB=Y | 0.553 | 0.119 | 4.636 | <0.001*** |
| Baseline PVR | 0.000 | 0.002 | 0.059 | 0.953 |
| Age at treatment start | 0.019 | 0.014 | 1.336 | 0.182 |
| Baseline Qmax | -0.015 | 0.028 | -0.515 | 0.607 |
| Baseline IPSS voiding subscore | -0.073 | 0.098 | -0.741 | 0.459 |
| Baseline prostate volume | -0.005 | 0.003 | -1.772 | 0.076 |
| TMTDG=Placebo | -1.000 | 0.246 | -4.067 | <0.001*** |
| MONTH | -0.321 | 0.027 | -11.698 | <0.001*** |
| MONTH’ | 2.514 | 0.367 | 6.842 | <0.001*** |
| MONTH’’ | -3.105 | 0.475 | -6.542 | <0.001*** |
| B_PVR*B_voiding_sub_score | 0.000 | 0.000 | 1.964 | 0.050* |
| AGE*B_voiding_sub_score | -0.004 | 0.001 | -3.089 | 0.002** |
| B_Qmax*B_voiding_sub_score | -0.005 | 0.003 | -1.777 | 0.076 |
| B_PV*TMTDG=Placebo | 0.016 | 0.004 | 4.086 | <0.001*** |
| TMTDG=Placebo*MONTH | 0.130 | 0.039 | 3.359 | 0.001** |
| TMTDG=Placebo*MONTH’ | -0.662 | 0.520 | -1.273 | 0.203 |
| TMTDG=Placebo*MONTH’’ | 0.809 | 0.672 | 1.205 | 0.228 |
| P-values have not been adjusted for multiplicity.  Interactions with associated p-values higher than 0.05 and insignificant improvement of the model have been excluded. | | | | |

*p-value between 0.01 and 0.05; **p-value between 0.001 and 0.01; ***p-value smaller than 0.001.
AB, alpha-blocker usage (yes/no) in the last 12 months; IPSS, International Prostate Symptom Score; PSA, prostate-specific antigen; PV, prostate volume; PVR, post-void residual urine; Qmax, maximum urinary flow rate; std error, standard error.

## Cross-validation and predictive capability

# Table S13. RMSE values from the 10-fold cross validation process. Placebo model voiding subscore

| Iteration number | RMSE  (train) | RMSE  (test) |
| --- | --- | --- |
| 1 | 3.590 | 3.544 |
| 2 | 3.571 | 3.710 |
| 3 | 3.600 | 3.436 |
| 4 | 3.565 | 3.770 |
| 5 | 3.597 | 3.477 |
| 6 | 3.586 | 3.587 |
| 7 | 3.592 | 3.515 |
| 8 | 3.603 | 3.420 |
| 9 | 3.567 | 3.741 |
| 10 | 3.573 | 3.697 |

RMSE, root mean squared error.

## Nocturia (IPSS Q7)

## CombAT model

Table S5 shows the AIC, BIC, and RMSE values from the GLS model without any interaction, as well as for the model obtained using the significant interactions among the baseline covariates and treatment, the baseline covariates, and baseline nocturia sub-score. Both models present very similar results for all the goodness of fit measures analyzed. The model with interactions has been used for the rest of the analysis for better understanding of the interactions between the covariates.

Table S14 shows a summary of the results from the final model. The estimated coefficients associated with each predictor and the relevant interactions are provided. Baseline Qmax, PVR, IPSS nocturia sub-score, age, and treatment with dutasteride are significantly associated with change. The positive sign of the coefficients indicates that higher values of PVR and age experiment a worsening on the symptoms of nocturia score. In the case of treatment with dutasteride, its positive coefficient (0.66) indicates that patients under the reference treatment (combination) are having a greater improvement. On the other hand, patients with high values of Qmax and IPSS nocturia sub-score at baseline, the opposite conclusion can be reached with higher baseline values leading to an improvement of the symptoms. Significant interactions effects between baseline IPSS nocturia sub-score and PVR as well as between AB or age with treatment are detected.

# Table S14. Estimates from the fitted model. CombAT model nocturia subscore

| **Summary coefficients** | | | | |
| --- | --- | --- | --- | --- |
|  | **Estimate** | **Std error** | **t-statistic** | **p-value** |
| Intercept | -0.035 | 0.190 | -0.185 | 0.853 |
| Baseline Qmax | -0.008 | 0.003 | -2.755 | 0.006** |
| Baseline prostate volume | 0.000 | 0.000 | 0.400 | 0.689 |
| Baseline PSA | -0.008 | 0.006 | -1.392 | 0.164 |
| Baseline PVR | 0.001 | 0.000 | 3.352 | 0.001** |
| Baseline IPSS Q7 nocturia | -0.595 | 0.019 | -31.571 | <0.001*** |
| Age at treatment start | 0.018 | 0.003 | 6.383 | <0.001*** |
| AB=Y | 0.010 | 0.046 | 0.214 | 0.831 |
| TMTDG=Dutasteride | 0.660 | 0.257 | 2.565 | 0.010** |
| TMTDG=Tamsulosin | 0.074 | 0.258 | 0.286 | 0.775 |
| MONTH | -0.010 | 0.002 | -4.484 | <0.001*** |
| MONTH’ | 0.017 | 0.009 | 1.933 | 0.053 |
| MONTH’’ | -0.028 | 0.020 | -1.403 | 0.161 |
| B_PVR*B_Q7 | -0.001 | 0.000 | -4.038 | <0.001*** |
| AGE*TMTDG=Dutasteride | -0.009 | 0.004 | -2.248 | 0.025** |
| AGE*TMTDG=Tamsulosin | -0.002 | 0.004 | -0.520 | 0.603 |
| AB=Y*TMTDG=Dutasteride | -0.003 | 0.065 | -0.040 | 0.968 |
| AB=Y*TMTDG=Tamsulosin | 0.172 | 0.065 | 2.625 | 0.009** |
| B_Q7*TMTDG=Dutasteride | 0.041 | 0.022 | 1.850 | 0.064 |
| B_Q7*TMTDG=Tamsulosin | 0.012 | 0.022 | 0.535 | 0.593 |
| TMTDG=Dutasteride*MONTH | -0.005 | 0.003 | -1.557 | 0.119 |
| TMTDG=Tamsulosin*MONTH | 0.006 | 0.003 | 1.864 | 0.062 |
| TMTDG=Dutasteride*MONTH’ | 0.015 | 0.012 | 1.230 | 0.219 |
| TMTDG=Tamsulosin*MONTH’ | 0.004 | 0.012 | 0.291 | 0.771 |
| TMTDG=Dutasteride*MONTH’’ | -0.034 | 0.028 | -1.183 | 0.237 |
| TMTDG=Tamsulosin*MONTH’’ | -0.014 | 0.029 | -0.500 | 0.617 |
| TMTDG=Dutasteride*MONTH’’ | -0.034 | 0.028 | -1.183 | 0.237 |
| TMTDG=Tamsulosin*MONTH’’ | -0.014 | 0.029 | -0.500 | 0.617 |
| P-values have not been adjusted for multiplicity.  Interactions with associated p-values higher than 0.05 and insignificant improvement of the model have been excluded. | | | | |

*p-value between 0.01 and 0.05; **p-value between 0.001 and 0.01; ***p-value smaller than 0.001.
AB, alpha-blocker usage (yes/no) in the last 12 months; IPSS, International Prostate Symptom Score; PSA, prostate-specific antigen; PVR, post-void residual urine; Qmax, maximum urinary flow rate; std error, standard error.

## Cross-validation and predictive capability

To test the predictive capability of the model with new data we performed a 10-fold cross-validation procedure.

# Table S15. RMSE values from the 10-fold cross validation process. CombAT model nocturia subscore

| **Iteration number** | **RMSE**  **(train)** | **RMSE**  **(test)** |
| --- | --- | --- |
| 1 | 1.007 | 1.042 |
| 2 | 1.015 | 0.968 |
| 3 | 1.012 | 0.99 |
| 4 | 1.011 | 0.998 |
| 5 | 1.005 | 1.063 |
| 6 | 1.011 | 1.002 |
| 7 | 1.009 | 1.021 |
| 8 | 1.013 | 0.986 |
| 9 | 1.012 | 0.991 |
| 10 | 1.004 | 1.064 |

RMSE, root mean squared error.

## Placebo model

Table S5 shows the AIC, BIC, and RMSE values from the GLS model without any interaction, as well as for the model obtained using the significant interactions among the baseline covariates and treatment, the baseline covariates, and baseline nocturia sub-score. Both models present very similar results for all the goodness of fit measures analyzed. The model with interactions has been used for the rest of the analysis for better understanding of the interactions between the covariates.

Table S16 shows the results from the final fitted model to the change of nocturia sub-scores for the studies of placebo and dutasteride. Baseline PSA, age, IPSS nocturia sub-score, and assignment to the placebo treatment group were all significant. Interactions between AB and IPSS nocturia sub-score as well as baseline PV and placebo treatment were also significant.

# Table S16. Estimates from the fitted model. Placebo model nocturia subscore

| **Summary coefficients** | | | | |
| --- | --- | --- | --- | --- |
| **Terms** | **Estimate** | **Std error** | **t-statistic** | **p-value** |
| Intercept | 0.986 | 0.125 | 7.899 | <0.001*** |
| Baseline PSA | -0.018 | 0.006 | -3.039 | 0.002** |
| Baseline PVR | 0.000 | 0.000 | 0.100 | 0.920 |
| Age at treatment start | 0.005 | 0.002 | 3.329 | 0.001** |
| Baseline Qmax | -0.006 | 0.003 | -1.698 | 0.090 |
| AB=Y | -0.029 | 0.077 | -0.372 | 0.710 |
| Baseline IPSS Q7 nocturia | -0.539 | 0.010 | -51.398 | <0.001*** |
| Baseline prostate volume | 0.000 | 0.001 | -0.641 | 0.522 |
| TMTDG=Placebo | -0.151 | 0.070 | -2.157 | 0.031* |
| MONTH | -0.050 | 0.009 | -5.479 | <0.001*** |
| MONTH’ | 0.440 | 0.122 | 3.622 | <0.001*** |
| MONTH’’ | -0.549 | 0.157 | -3.493 | 0.001** |
| AB=Y*B_Q7 | 0.079 | 0.028 | 2.787 | 0.005** |
| B_PV*TMTDG=Placebo | 0.003 | 0.001 | 2.595 | 0.010** |
| TMTDG=Placebo*MONTH | 0.013 | 0.013 | 1.015 | 0.310 |
| TMTDG=Placebo*MONTH’ | -0.036 | 0.172 | -0.212 | 0.832 |
| TMTDG=Placebo*MONTH’’ | 0.043 | 0.222 | 0.192 | 0.848 |
| P-values have not been adjusted for multiplicity.  Interactions with associated p-values higher than 0.05 and insignificant improvement of the model have been excluded. | | | | |

*p-value between 0.01 and 0.05; **p-value between 0.001 and 0.01; ***p-value smaller than 0.001.
AB, alpha-blocker usage (yes/no) in the last 12 months; IPSS, International Prostate Symptom Score; PSA, prostate-specific antigen; PV, prostate volume; PVR, post-void residual urine; Qmax, maximum urinary flow rate; std error, standard error.

## Cross-validation and predictive capability

# Table S17. RMSE values from the 10-fold cross validation process. Placebo model nocturia subscore

| **Iteration number** | **RMSE  (train)** | **RMSE**  **(test)** |
| --- | --- | --- |
| 1 | 1.068 | 1.114 |
| 2 | 1.076 | 1.041 |
| 3 | 1.075 | 1.053 |
| 4 | 1.072 | 1.074 |
| 5 | 1.074 | 1.055 |
| 6 | 1.075 | 1.050 |
| 7 | 1.074 | 1.056 |
| 8 | 1.068 | 1.110 |
| 9 | 1.073 | 1.070 |
| 10 | 1.068 | 1.109 |

RMSE, root mean squared error.

## IPSS Q8

## CombAT model

Table S5 shows the AIC, BIC, and RMSE values from the GLS model without any interaction, as well as for the model obtained using the significant interactions among the baseline covariates and treatment, the baseline covariates, and baseline IPSS quality of life (QoL) sub-score. Both models present very similar results for all the goodness of fit measures analyzed. The model with interactions has been used for the rest of the analysis for better understanding of the interactions between the covariates.

Table S18 shows the estimated parameters, standard errors, and p-values. The baseline covariates PVR, IPSS QoL sub-score, AB, and total IPSS are significant predictors of the IPSS QoL change. The interaction of treatment with IPSS QoL sub-score, PV, age, and Qmax is also significant.

# Table S18. Estimates from the fitted model. CombAT model IPSS Q8 QoL subscore

| **Summary coefficients** | | | | |
| --- | --- | --- | --- | --- |
|  | **Estimate** | **Std error** | **t-statistic** | **p-value** |
| Intercept | 1.489 | 0.277 | 5.370 | <0.001*** |
| Baseline PVR | 0.001 | 0.000 | 2.600 | 0.009** |
| Baseline Qmax | -0.022 | 0.011 | -2.110 | 0.035* |
| Baseline IPSS Q8 (QoL) | -0.800 | 0.037 | -21.710 | <0.001*** |
| AB=Y | 0.076 | 0.034 | 2.230 | 0.026* |
| Baseline total IPSS | 0.021 | 0.003 | 7.920 | <0.001*** |
| Age at treatment start | 0.003 | 0.004 | 0.780 | 0.438 |
| Baseline prostate volume | -0.001 | 0.001 | -0.700 | 0.483 |
| Baseline PSA | 0.006 | 0.012 | 0.500 | 0.620 |
| TMTDG=Dutasteride | 0.690 | 0.347 | 1.990 | 0.047 |
| TMTDG=Tamsulosin | 0.344 | 0.346 | 0.990 | 0.320 |
| MONTH | -0.042 | 0.002 | -18.450 | <0.001*** |
| MONTH’ | 0.077 | 0.009 | 8.580 | <0.001*** |
| MONTH’’ | -0.142 | 0.021 | -6.800 | <0.001*** |
| B_Qmax*B_QOL | 0.006 | 0.003 | 2.070 | 0.039* |
| AGE*TMTDG=Dutasteride | -0.008 | 0.005 | -1.650 | 0.099 |
| AGE*TMTDG=Tamsulosin | -0.010 | 0.005 | -1.990 | 0.047* |
| B_PV*TMTDG=Dutasteride | 0.001 | 0.002 | 0.500 | 0.620 |
| B_PV*TMTDG=Tamsulosin | 0.004 | 0.002 | 2.370 | 0.018* |
| B_PSA*TMTDG=Dutasteride | -0.033 | 0.017 | -1.870 | 0.061 |
| B_PSA*TMTDG=Tamsulosin | -0.017 | 0.018 | -0.960 | 0.337 |
| B_QOL*TMTDG=Dutasteride | 0.077 | 0.027 | 2.870 | 0.004** |
| B_QOL*TMTDG=Tamsulosin | 0.041 | 0.027 | 1.510 | 0.131 |
| TMTDG=Dutasteride*MONTH | -0.007 | 0.003 | -2.060 | 0.039* |
| TMTDG=Tamsulosin*MONTH | 0.019 | 0.003 | 5.890 | <0.001*** |
| TMTDG=Dutasteride*MONTH’ | 0.013 | 0.013 | 1.040 | 0.299 |
| TMTDG=Tamsulosin*MONTH’ | -0.030 | 0.013 | -2.320 | 0.021* |
| TMTDG=Dutasteride*MONTH’’ | -0.025 | 0.030 | -0.840 | 0.399 |
| TMTDG=Tamsulosin*MONTH’’ | 0.056 | 0.030 | 1.880 | 0.060 |
| P-values have not been adjusted for multiplicity.  Interactions with associated p-values higher than 0.05 and insignificant improvement of the model have been excluded. | | | | |

*p-value between 0.01 and 0.05; **p-value between 0.001 and 0.01; ***p-value smaller than 0.001.
AB, alpha-blocker usage (yes/no) in the last 12 months; IPSS, International Prostate Symptom Score; PSA, prostate-specific antigen; PV, prostate volume; PVR, post-void residual volume; Qmax, maximum urinary flow rate; QoL, quality of life; std error, standard error.

## Cross-validation and predictive capability

# Table S19. RMSE values from the 10-fold cross validation process. CombAT model IPSS Q8 QoL sub-score

| **Iteration number** | **RMSE**  **(train)** | **RMSE**  **(test)** |
| --- | --- | --- |
| 1 | 1.173 | 1.188 |
| 2 | 1.174 | 1.173 |
| 3 | 1.176 | 1.153 |
| 4 | 1.176 | 1.157 |
| 5 | 1.175 | 1.166 |
| 6 | 1.17 | 1.213 |
| 7 | 1.176 | 1.167 |
| 8 | 1.174 | 1.17 |
| 9 | 1.173 | 1.178 |
| 10 | 1.17 | 1.21 |

IPSS, International Prostate Symptom Score; RMSE, root mean squared error; QoL, quality of life.

## Benign prostatic hyperplasia impact index

## CombAT model

Table S5 shows the AIC, BIC, and RMSE values from the GLS model without any interaction, as well as for the model obtained using the significant interactions among the baseline covariates and treatment, the baseline covariates, and baseline benign prostatic hyperplasia impact index (BII) score. Both models present very similar results for all the goodness of fit measures analyzed. The model with interactions has been used for the rest of the analysis for better understanding of the interactions between the covariates.

Table S20 shows the estimated parameters, standard errors, and p-values. The baseline covariates age, PVR, BII score, total IPSS, and treatment are significant predictors of the BII change score. Significant interaction of treatment with AB and time is found.

# Table S20. Estimates from the fitted model. CombAT model BII score

| **Summary coefficients** | | | | | |
| --- | --- | --- | --- | --- | --- |
|  | **Estimate** | **Std error** | **t-statistic** | | **p-value** |
| Intercept | 2.091 | 0.319 | 6.560 | <0.001*** | |
| Baseline PVR | 0.001 | 0.000 | 2.070 | 0.039* | |
| Baseline Qmax | -0.014 | 0.008 | -1.760 | 0.079 | |
| Baseline prostate volume | -0.001 | 0.001 | -0.710 | 0.477 | |
| Age at treatment start | -0.009 | 0.004 | -2.270 | 0.023* | |
| Baseline BPH Impact Index Score | -0.575 | 0.011 | -52.340 | <0.001*** | |
| Baseline total IPSS | 0.017 | 0.006 | 3.060 | 0.002** | |
| Baseline PSA | 0.024 | 0.024 | 0.970 | 0.333 | |
| AB=Y | -0.126 | 0.120 | -1.050 | 0.293 | |
| TMTDG=Dutasteride | 0.694 | 0.163 | 4.250 | <0.001*** | |
| TMTDG=Tamsulosin | -0.070 | 0.164 | -0.430 | 0.669 | |
| MONTH | -0.060 | 0.005 | -13.270 | <0.001*** | |
| MONTH’ | 0.106 | 0.018 | 6.010 | <0.001*** | |
| MONTH’’ | -0.185 | 0.041 | -4.470 | <0.001*** | |
| B_PSA*TMTDG=Dutasteride | -0.058 | 0.034 | -1.720 | 0.085 | |
| B_PSA*TMTDG=Tamsulosin | -0.012 | 0.034 | -0.360 | 0.717 | |
| AB=Y*TMTDG=Dutasteride | 0.231 | 0.168 | 1.370 | 0.170 | |
| AB=Y*TMTDG=Tamsulosin | 0.462 | 0.170 | 2.720 | 0.007** | |
| TMTDG=Dutasteride*MONTH | -0.013 | 0.006 | -2.100 | 0.036* | |
| TMTDG=Tamsulosin*MONTH | 0.030 | 0.006 | 4.710 | <0.001*** | |
| TMTDG=Dutasteride*MONTH’ | 0.030 | 0.025 | 1.190 | 0.235 | |
| TMTDG=Tamsulosin*MONTH’ | -0.016 | 0.025 | -0.640 | 0.525 | |
| TMTDG=Dutasteride*MONTH’’ | -0.062 | 0.058 | -1.070 | 0.285 | |
| TMTDG=Tamsulosin*MONTH’’ | 0.004 | 0.059 | 0.070 | 0.946 | |
| P-values have not been adjusted for multiplicity.  Interactions with associated p-values higher than 0.05 and insignificant improvement of the model have been excluded. | | | | | |

*p-value between 0.01 and 0.05; **p-value between 0.001 and 0.01; ***p-value smaller than 0.001.
AB, alpha-blocker usage (yes/no) in the last 12 months; BII, benign prostatic hyperplasia impact index; BPH, benign prostatic hyperplasia; IPSS, International Prostate Symptom Score; PSA, prostate-specific antigen; PVR, post-void residual volume; Qmax, maximum urine flow rate; std error, standard error.

## Cross-validation and predictive capability

# Table S21. RMSE values from the 10-fold cross validation process. CombAT model BII score

| **Iteration number** | **RMSE**  **(train)** | **RMSE**  **(test)** |
| --- | --- | --- |
| 1 | 2.374 | 2.379 |
| 2 | 2.372 | 2.375 |
| 3 | 2.376 | 2.355 |
| 4 | 2.377 | 2.345 |
| 5 | 2.374 | 2.375 |
| 6 | 2.375 | 2.37 |
| 7 | 2.38 | 2.324 |
| 8 | 2.37 | 2.405 |
| 9 | 2.365 | 2.446 |
| 10 | 2.37 | 2.412 |

BII, benign prostatic hyperplasia impact index; RMSE, root mean squared error.

## Placebo model

Table S5 shows the AIC, BIC, and RMSE values from the GLS model without any interaction, as well as for the model obtained using the significant interactions among the baseline covariates and treatment, the baseline covariates, and baseline BII score. Both models present very similar results for all the goodness of fit measures analyzed. The model with interactions has been used for the rest of the analysis for better understanding of the interactions between the covariates.

Table S22 shows the results from the final fitted model to the change of BII scores for the studies of placebo and dutasteride. Baseline values of PSA, AB, total IPSS, treatment, and time were significant predictors in the model. Interactions between QT and age and QT and PVR were significant, as was an interaction between PV and treatment.

# Table S22. Estimates from the fitted model. Placebo model BII score

| **Summary coefficients** | | | | |
| --- | --- | --- | --- | --- |
|  | **Estimate** | **Std error** | **t-statistic** | **p-value** |
| Intercept | 1.079 | 0.454 | 2.380 | 0.017* |
| Baseline Qmax | -0.012 | 0.008 | -1.510 | 0.130 |
| Baseline PSA | -0.032 | 0.013 | -2.420 | 0.015* |
| Baseline BPH Impact Index Score | -0.171 | 0.088 | -1.940 | 0.053 |
| AB=Y | 0.208 | 0.073 | 2.840 | 0.005** |
| Baseline total IPSS | 0.047 | 0.005 | 8.830 | <0.001*** |
| Age at treatment start | 0.005 | 0.006 | 0.740 | 0.462 |
| Baseline PVR | -0.001 | 0.001 | -0.810 | 0.416 |
| Baseline prostate volume | -0.002 | 0.002 | -1.070 | 0.284 |
| TMTDG=Placebo | -0.613 | 0.151 | -4.060 | <0.001*** |
| MONTH | -0.136 | 0.017 | -7.930 | <0.001*** |
| MONTH’ | 0.821 | 0.230 | 3.580 | <0.001*** |
| MONTH’’ | -0.975 | 0.297 | -3.290 | 0.001*** |
| B_QT*AGE | -0.005 | 0.001 | -3.460 | 0.001*** |
| B_QT*B_PVR | 0.000 | 0.000 | 2.770 | 0.006** |
| B_PV*TMTDG=Placebo | 0.009 | 0.002 | 3.640 | <0.001*** |
| TMTDG=Placebo*MONTH | 0.074 | 0.024 | 3.040 | 0.002** |
| TMTDG=Placebo*MONTH’ | -0.279 | 0.325 | -0.860 | 0.390 |
| TMTDG=Placebo*MONTH’’ | 0.326 | 0.420 | 0.780 | 0.438 |
| P-values have not been adjusted for multiplicity.  Interactions with associated p-values higher than 0.05 and insignificant improvement of the model have been excluded. | | | | |

*p-value between 0.01 and 0.05; **p-value between 0.001 and 0.01; ***p-value smaller than 0.001.
AB, alpha-blocker usage (yes/no) in the last 12 months; BII, benign prostatic hyperplasia impact index; BPH, benign prostatic hyperplasia; IPSS, International Prostate Symptom Score; PSA, prostate specific antigen; PV, prostate volume; PVR, post-void residual volume; Qmax, maximum urinary flow rate; std error, standard error.

## Cross-validation and predictive capability

# Table S23. RMSE values for the 10-fold cross validation process. Placebo model BII score

| **Iteration number** | **RMSE**  **(train)** | **RMSE**  **(test)** |
| --- | --- | --- |
| 1 | 2.198 | 2.109 |
| 2 | 2.202 | 2.064 |
| 3 | 2.193 | 2.16 |
| 4 | 2.188 | 2.198 |
| 5 | 2.196 | 2.133 |
| 6 | 2.185 | 2.229 |
| 7 | 2.189 | 2.196 |
| 8 | 2.183 | 2.239 |
| 9 | 2.186 | 2.214 |
| 10 | 2.168 | 2.368 |

BII, benign prostatic hyperplasia impact index; RMSE, root mean squared error.

# Supplementary references

1. Gravas S, Palacios-Moreno JM, Thompson D, Concas F, Kamola PJ, Roehrborn CG, et al. Understanding treatment response in individual profiles of men with prostatic enlargement at risk of progression. Eur Urol Focus. 2023;9:178–87.
2. Harrell Jr FE. Regression modeling strategies: with applications to linear models, logistic and ordinal regression, and survival analysis. 2nd ed. Cham: Springer; 2015.
3. Steyerberg EW, Eijkemans MJ, Habbema JD. Application of shrinkage techniques in logistic regression analysis: a case study. Stat Neerl. 2001 Mar;55:76–88.
4. Roehrborn CG, Boyle P, Nickel JC, Hoefner K, Andriole G. Efficacy and safety of a dual inhibitor of 5-alpha-reductase types 1 and 2 (dutasteride) in men with benign prostatic hyperplasia. Urology*.* 2002;60:434–41.
5. Roehrborn CG, Siami P, Barkin J, Damiao R, Major-Walker K, Nandy I, et al. The effects of combination therapy with dutasteride and tamsulosin on clinical outcomes in men with symptomatic benign prostatic hyperplasia: 4-year results from the CombAT study. Eur Urol. 2010;57:123–31.
6. Committee for Human Medicinal Products. Addendum on Estimands and Sensitivity Analysis in Clinical Trials To the Guideline on Statistical Principles for Clinical Trials E9(R1). London: European Medicines Evaluation Agency; 2019. Available from: <https://database.ich.org/sites/default/files/E9-R1_Step4_Guideline_2019_1203.pdf>
7. Steyerberg EW. Clinical Prediction Models: A practical approach to development, validation, and updating. 1st ed. Cham: Springer; 2009.
8. Vergowe Y, Royston P, Moons KGM, Altman DG. Development and validation of a prediction model with missing predictor data: a practical approach. J Clin Epidemiol. 2010;63:205–14.
